# Ruthenium Complexes in the Fight against Pathogenic Microorganisms. An Extensive Review

**DOI:** 10.3390/pharmaceutics13060874

**Published:** 2021-06-13

**Authors:** Alexandra-Cristina Munteanu, Valentina Uivarosi

**Affiliations:** Department of General and Inorganic Chemistry, Faculty of Pharmacy, “Carol Davila” University of Medicine and Pharmacy, 020956 Bucharest, Romania

**Keywords:** ruthenium, antimicrobial, antibacterial, antiviral, antiparasitic, COVID-19

## Abstract

The widespread use of antibiotics has resulted in the emergence of drug-resistant populations of microorganisms. Clearly, one can see the need to develop new, more effective, antimicrobial agents that go beyond the explored ‘chemical space’. In this regard, their unique modes of action (e.g., reactive oxygen species (ROS) generation, redox activation, ligand exchange, depletion of substrates involved in vital cellular processes) render metal complexes as promising drug candidates. Several Ru (II/III) complexes have been included in, or are currently undergoing, clinical trials as anticancer agents. Based on the in-depth knowledge of their chemical properties and biological behavior, the interest in developing new ruthenium compounds as antibiotic, antifungal, antiparasitic, or antiviral drugs has risen. This review will discuss the advantages and disadvantages of Ru (II/III) frameworks as antimicrobial agents. Some aspects regarding the relationship between their chemical structure and mechanism of action, cellular localization, and/or metabolism of the ruthenium complexes in bacterial and eukaryotic cells are discussed as well. Regarding the antiviral activity, in light of current events related to the Covid-19 pandemic, the Ru (II/III) compounds used against SARS-CoV-2 (e.g., BOLD-100) are also reviewed herein.

## 1. Introduction

The alarming pace at which microorganisms are evading antibiotics constitutes a challenge for modern medicine [1]. The phenomenon of multidrug resistance has generated a sense of urgency around the development of new classes of antibiotics. Yet most of the drugs under clinical development for the treatment of bacterial infections are organic derivatives of currently used antibiotics, which suggests that these molecules are susceptible to in place mechanisms of bacterial resistance [2].

Although the pipeline for new antibiotics is running dry, the coordination chemistry field is still largely underexplored for antibacterial drug development, with limited clinical use for bismuth and silver-based antimicrobials. Bismuth compounds, for instance, are used for the treatment of *H. pylori* infections and diarrhea and in wound dressings [3], while silver compounds are used for wound healing applications and management of topical infections [4]. The focus of current research is directed towards the development of metal-based nanoparticles (NPs), with special interest being given to AgNPs following their introduction to the U.S. market in 2016 [5]. 

It is rather unfortunate that less attention is being given to metal complexes. It should be noted that metal-based compounds offer a vast structural diversity of three-dimensional (3D) scaffolds due to the variety of metal ions, ligands, and possible geometries [2,6,7]. While most organic fragments have linear (1D) or planar (2D) shapes, more complex 3D fragments are desirable for the molecular recognition by biomolecules and optimal interaction with intracellular targets [6]. Furthermore, increasing the 3D chemical topology of molecules has been correlated with a broader activity spectrum [7,8]. Therefore, metal complexes are ideal candidates for future drug discovery pursuits meant to access the underexplored 3D chemical space [6]. In addition, metal complexes possess unique mechanisms of action that are not readily available to organic compounds: ROS generation, redox activation, ligand exchange, and depletion of substrates involved in vital cellular processes [2,9,10]. When compared with solely organic molecules, metal-based compounds were found to display a significantly higher hit-rate against critical antibiotic-resistant pathogens (0.87% vs. 9.9%). Moreover, the percentages of toxic to healthy eukaryotic cells and/or hemolytic compounds in the two groups were found to be nearly identical. Therefore, a generally higher degree of toxicity cannot explain the remarkably high antimicrobial activity of the metal-based set of compounds compared with the organic molecules [2].

The potential of metal complexes has been acknowledged over the last two decades through several platinum-, ruthenium-, copper-, iron-, and gallium-based drugs, which have reached different stages in clinical trials for the treatment of cancer, neurodegenerative diseases, and malaria [11,12]. Several ruthenium (Ru) complexes have been evaluated in clinical trials for the treatment of cancer, namely NAMI-A [13,14], KP1019 [15,16] and its water-soluble sodium salt IT-139 (formerly KP1339) [17], and, more recently, TLD-1433 [18]. Previous knowledge of their chemical properties and biological behavior, gained from the research directed towards the development of novel anticancer compounds, has led to increased focus on tailoring ruthenium complexes as antimicrobial agents [1]. Moreover, a recent study screening 906 metal-containing compounds for antimicrobial activity identified ruthenium as the most frequent element found in active compounds that are nontoxic to eukaryotic cells, followed by silver, palladium, and iridium [2]. Therefore, ruthenium-based compounds hold promise for potential antimicrobial applications, which will be extensively reviewed in this paper.

In order to clarify the use of the terms ‘antibacterial’, ‘antibiotic’, and ‘antimicrobial’ in this manuscript, definitions are given below. The term antibacterial refers to substances, materials, or assemblies that kill or inhibit the growth of bacteria. WHO defines an antibiotic as a substance with a direct action on bacteria that is used for the treatment or prevention of infections or infectious diseases [19]. Although we recognize the distinction between these two terms, in order to avoid repetition, we have occasionally used the terms ‘antibiotic’ and ‘antibacterial’ interchangeably. Antimicrobials, on the other hand, will be used generically for compounds or materials that act against microorganisms (bacteria, fungi, viruses, protozoa, parasites, etc.). Consequently, antimicrobials will include antibacterials, antifungals, antivirals, antiprotozoals, and antiparasitics.

## 2. General Remarks on Bacterial Cell Structure. Gram-Positive vs. Gram-Negative Strains

The bacterial cell structure comes as a result of the extreme conditions they must survive in, which are inhospitable for eukaryotes. For instance, the rigid cell wall that covers the cell membrane is vital for protection from physical, chemical, and mechanical stressors. Based on the Gram staining procedure, bacteria are classified into two groups: Gram-positive and Gram-negative bacteria [1].

**Figure 1:**
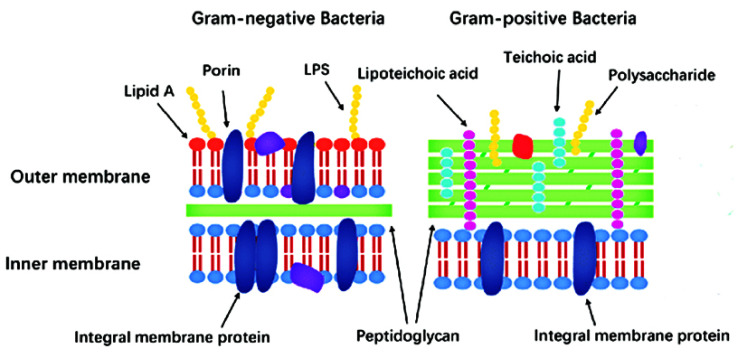
Comparison between Gram-negative and Gram-positive bacteria cell walls. Adapted from [22] with permission. Copyright © 2020 Huan, Kong, Mou and Yi.

Gram-positive strains retain the Crystal Violet stain due to the presence of a thick layer of peptidoglycan in their cell walls, which is densely embedded with negatively charged glycopolymers called wall teichoic acids (Figure 1). The fairly porous cell wall structure generally allows for passage for exogenous molecules into the bacterial cells [20].

Gram-negative bacteria, however, have more complex cell wall structures (Figure 1). Due to the absence of inlaid teichoic acid molecules, their layer of peptidoglycan is thin, yet bound to an outer membrane coated with lipopolysaccharides (LPSs). LPSs are amphiphiles, consisting of a hydrophobic lipidic domain (lipid A) covalently bound to a polysaccharide, which comprises the O antigen and the inner and outer cores; these negatively charged (due to the presence of the phosphate and acid groups) macromolecules are stabilized by divalent cations such as calcium and magnesium. LPSs greatly decrease bacterial permeability to antibiotics and play a crucial role in the development of resistance mechanisms for many pathogenic Gram-negative bacteria [1,20].

Additionally, on the cell surface of some bacteria (e.g., *Streptococcus pneumoniae*) a slime layer or a capsule can offer additional protection against desiccation or phagocytosis by host cells. Flagella, fimbriae, and pili are external filamentous appendages that serve as organelles of locomotion or assist with bacterial attachment and adhesion to a surface or genetic exchange [1,21].

At physiological pH, the high content of zwitterionic phosphatidylcholine confers an overall neutral charge to the eukaryotic cell membranes. In contrast, bacterial outer cell walls and membranes are usually negatively charged due to the presence of negatively charged components (phospholipids, teichoic acids, and lipopolysaccharides) [1,23]. Hence, in order to increase selectivity, new antibacterial drugs (including ruthenium complexes) are generally designed so as to possess a cationic component.

## 3. Mechanisms of Action of Current Drugs

**Figure 2:**
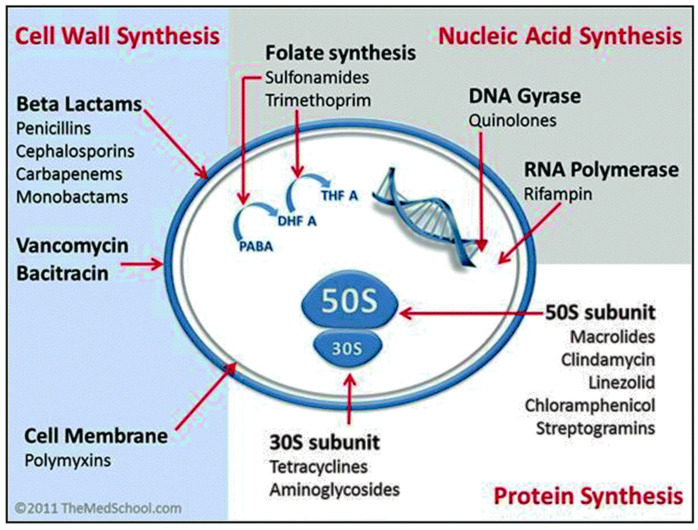
Mechanisms of action of currently used antibiotics (Image by Kendrick Johnson, licensed under the Creative Commons Attribution-Share Alike 3.0 Unported license).

Antibiotics are classified into four major groups (Figure 2), based on their intracellular target and mechanism of action: (1) inhibition of bacterial cell wall synthesis (penicillin and its derivatives, cephalosporins, carbapenems, and glycopeptides—these drugs are more active against Gram-positive bacteria); (2) disruption of bacterial membranes (polymyxins—these are active against Gram-negative bacteria and considered a last-line therapy against Gram-negative ‘superbugs’); (3) inhibition of nucleic acid synthesis (quinolones, rifampicin, and sulphonamidesare—these are broad-spectrum synthetic antibiotics); and (4) inhibition of protein synthesis (tetracycline, aminoglycosides, chloramphenicol, and macrolides—these inhibit protein synthesis by targeting the RNA-rich surfaces of ribosomes) [1].

Several new classes of antibiotics have been discovered over the last two decades. Gepotidacin, for instance, belongs to a new chemical class of antibiotics called triazaacenaphthylene. It is a topoiosomerase inhibitor, which is currently being investigated in a phase III clinical study in patients with uncomplicated urinary tract infection and urogenital gonorrhoea [24]. Other current strategies include the use of phages (viruses that kill specific bacterial strains) [25], various types of engineered nanoparticles [25], and cationic materials, including cationic polypeptides, polymers, copolymers, and dendrimers [26]. Furthermore, several natural products, e.g., teixobactin, have been identified as lead compounds in the fight against antimicrobial resistance [27].

## 4. Mechanisms of Resistance to Antibiotics

Bacterial resistance to antibiotics can result from intrinsic or acquired antibiotic-resistant mechanisms. *P. aeruginosa* and other Gram-negative pathogens are intrinsically more resistant to antibiotics due to the reduced permeability of their outer membranes. These bacterial strains have porins of unusually low permeability. In addition, the outer membranes of mycobacteria have a high lipid content that allows for hydrophobic drugs such as fluoroquinolones to enter the cell but limits the access of hydrophilic drugs.

Acquired bacterial resistance is caused by alterations in microorganisms that result in drug inactivation or a decrease in therapeutic efficacy. Improper prescribing and overuse of antibiotics are factors that have contributed to the growing issue of microbial resistance. Consequently, infections have become increasingly difficult or even impossible to treat [28].

Bacterial resistance can emerge as a result of various biochemical mechanisms, including decreased drug uptake, modification of a specific bacterial target, enzymatic inactivation of the drug, and modifications to the bacterial efflux systems [1,28]. For instance, a common resistance mechanism is the alteration of the bacterial membrane permeability, resulting in limited uptake of an antibiotic. Modification of the drug’s target can involve mutations in DNA gyrase and topoisomerase IV or alterations in the structure and/or number of penicillin-binding proteins [5]. Drug inactivation occurs via mutations in genes coding for key enzymes, such as β-lactamases, acetyltransferases, adenylyltransferases, and aminoglycoside-3′-phosphotransferase. These mutations can occur either inside the bacterial chromosomal DNA or as a result of foreign genetic material acquisition. Acquisition of genetic material that confers resistance is possible through horizontal gene transfer, which is mediated either by plasmids or bacteriophages [28].

Another common mechanism of resistance used by many pathogens involves the association of multiple bacterial cells in matrices called biofilms. The bacterial cells within the biofilm have a slow metabolism rate and slow cell division. Therefore, antimicrobials targeting growing and dividing bacterial cells are rendered ineffective. Moreover, the thick biofilm extracellular matrix consists of bacterial polysaccharides, proteins, and DNA, which hinder access of the antimicrobial agent to the bacteria. It is also likely that the proximity of the bacterial cells facilitates horizontal gene transfer. Therefore, the antimicrobial resistance genes can be shared between the cells forming the biofilm [28,29,30].

Nosocomial infections or hospital-acquired infections are a growing threat worldwide and are often caused by multidrug-resistant bacteria. Interestingly, a small group of microorganisms, known as ESKAPE pathogens, are responsible for most antibiotic-resistant infections. These pathogens include: *Enterococcus faecium, Staphylococcus aureus, Klebsiella pneumoniae, Acinetobacter baumannii, Pseudomonas aeruginosa*, and *Enterobacter* spp., which possess innate resistance or can acquire resistance against multiple antibiotics [31].

## 5. Antibacterial and Antifungal Activities of Ruthenium Complexes

Based upon their chemical stability, Ru complexes can be classified as either stable, relatively inert compounds, and prodrugs. A metal complex is inert when the ligand framework remains unaltered in biological media. The ruthenium ion in these compounds acts merely as a central scaffold that carries the bioactive ligands to their target. Consequently, the properties of the coordinated ligands are essential to the antibacterial activity [32]. The presence of the ruthenium ion, however, provides the molecule with a positive charge, which aids in targeting the negatively charged cell wall structures of bacteria. The antibacterial activity of these complexes depends on their lipophilicity and charge, which in turn shape their ability to interact with specific targets (e.g., DNA, RNA, proteins, bacterial membranes).

Prodrugs are labile complexes that release the ligand/s when exposed to solvents and/or media and generate species that can bind to various biological targets or photoactivated drugs. The latter become active upon light irradiation and act as photosensitizers. Since this behavior is somewhat unconventional for the general understanding of the term ‘prodrug’ in the traditional medicinal chemistry sense, ‘prodrug-like molecules’ seems more appropriate to describe this type of metal complex. In the case of labile complexes, active species are released as a result of either partial or total ligand exchange in biological media. These active species are either ruthenium species resulting from ligand exchange with media components or the released ligands. In the latter case, the ruthenium compounds are called ‘carrier’ complexes; one such example is the Ru(II) chelate–chloroquine complex, [RuCl_2_(CQ)]_2_, where CQ = chloroquine (see 6. Antiparasitic activity of ruthenium complexes). In the following sections, ruthenium complexes will be classified based on their structure. Details and comments with regard to their mechanisms of action will be provided wherever such information is available.

### 5.1. Mononuclear Ruthenium (II) Complexes

Mononuclear polypyridylruthenium (II) complexes with antimicrobial activities were first reported in the 1950s and 1960s by Dwyer et al. [33,34]. With the general interest shifting towards discovering new analogues of existing classes of antibiotics, their impressive seminal work was unfortunately not further pursued. However, the advancement into clinical trials of NAMI-A, KP1019, and TLD1433 for the treatment of cancer and the urge to develop new classes of antibiotics have led, over the last two decades, to an increased focus on research and development of ruthenium-based antimicrobials [35].

**Figure 3:**
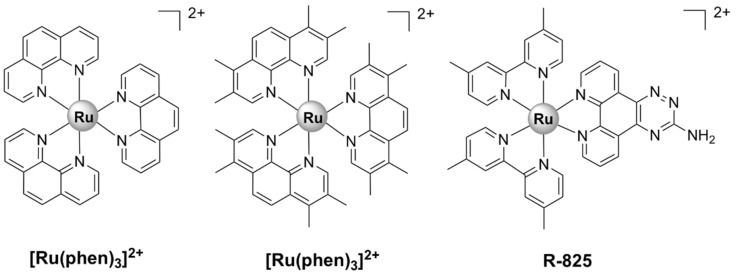
Examples of inert structural mononuclear polypyridylruthenium (II) complexes.

Dwyer et al. made the first steps towards the development of kinetically inert Ru(II) complexes and the study of their in vitro and in vivo antimicrobial activities. The addition of methyl groups to the phenanthroline ligands enhanced lipophilicity and increased the activity of [Ru(Me_4_phen)_3_]^2+^ (Figure 3) against Gram-positive bacteria, as compared with [Ru(phen)_3_]^2+^ (Figure 3) [36]. More recent studies [37,38], however, have shown that these complexes are much less active against various antibiotic-resistant ESKAPE pathogens. Additionally, their activity in vivo has been proven to be unsatisfactory, as they caused severe neurotoxic effects when injected into mice [39].

Following up on this remarkable work, various heteroleptic mononuclear polypyridyl Ru (II) complexes were tested for antibacterial activity. Their activities (MIC values) against various bacterial strains, as well as toxicity towards healthy eukaryotic cells and modes of action, where available, are listed in Table 1.

#### 5.1.1. Mononuclear Polypyridyl Ru (II) Complexes

R-825 (Figure 3) was shown to interfere with the iron acquisition systems in *S. pneumoniae*, which led to a dramatic decrease in intracellular iron, correlated with a bactericidal effect. In addition, R-825 was essentially non-toxic to human A549 non-small-cell lung cancer cells in vitro [41]. Iron is an essential nutrient for the development and survival of bacteria, as well as a key factor in host infection. In order to scavenge iron from their surroundings, bacteria make use of highly effective iron acquisition systems. In *S. pneumoniae*, the ABC transporters PiaABC, PiuABC, and PitABC play a major role in the acquisition of heme, ferrichrome, and ferric irons, respectively [72]. The deletion of the *piuA* gene in a mutant strain of *S. pneumoniae* resulted in a significant decrease in ruthenium uptake, leading to an increased resistance of the mutant to R-825 treatment. These results suggest that the mechanism of uptake for R-825 appears to involve active transport via the PiuABC iron uptake pathway [41]. Note that this mechanism of uptake is different than those used by the currently approved antibiotics. Generally, due to the chemical similarity between iron and ruthenium, the ability of novel antibiotics to interfere with iron acquisition systems in bacteria (including ABC transporters) is considered to be a viable strategy for the discovery of new antibacterial drugs.

A variety of mononuclear heteroleptic polypyridyl ruthenium (II) chelates bearing bpy, phen, dmp (4,4′-dimethyl-2,2′-bipyridine), or hdpa (2,2’-dipyridylamine) and other mono/bidentate ligands were active in various degrees against Gram-positive and Gram-negative bacteria and fungi [73,74,75,76,77,78,79,80,81]. Although their mechanisms of action have not been determined, all complexes were shown to interact with DNA duplexes and several exerted photoactivated cleavage of plasmid DNA in vitro [75,77,79,80,81] with singlet oxygen (^1^O_2_) probably playing a significant role in the cleavage mechanism.

##### Mononuclear Ru(II) Heteroleptic Complexes Bearing 2,2’-Bipyridine (bpy) Ligands

**Figure 4:**
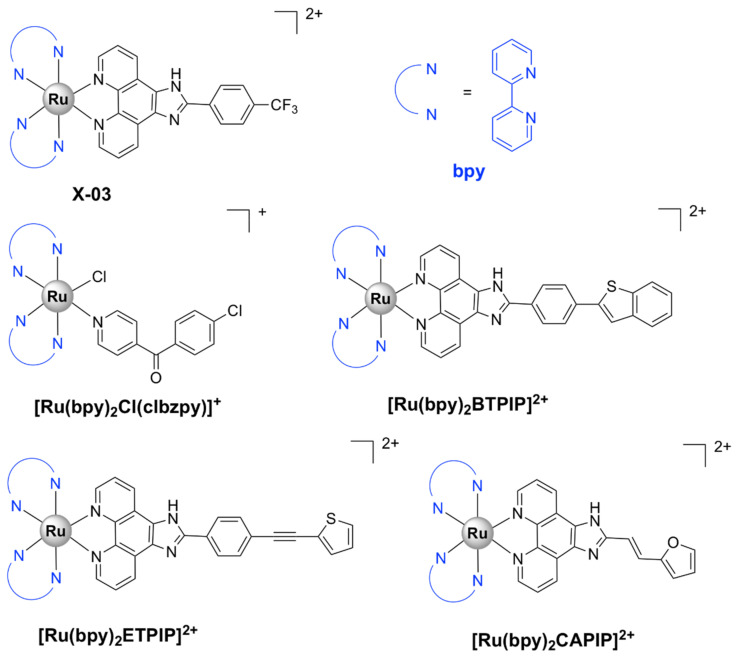
Chemical structures of heteroleptic Ru(II) complexes bearing 2,2’-bipyridine (bpy) ligands. BTPIP = (2-(4-(benzo[b]thiophen-2-yl)phenyl)-1*H*-imidazo [4,5-*f*][1,10]phenanthroline); ETPIP = 2-(4-(thiophen-2-ylethynyl)phenyl)-1*H*-imidazo[4,5-*f*][1,10]phenanthroline); CAPIP = *(E)*-2-(2-(furan-2-yl)vinyl)-1*H*-imidazo[4,5-*f*][1,10]phenanthroline; dmp = 4,4’-dimethyl-2,2’-bipyridine; bpy = 2,2’-bipyridine; phen = 1,10-phenanthroline.

Numerous octahedral heteroleptic Ru(II) complexes containing 2,2’-bipyridine (bpy), with the general formula [Ru(bpy)_2_L]Y_n_ (where L = a mono/bidentate ligand, note that when L is monodentate, the first coordination sphere of Ru(II) is saturated with chloride ions; Y = counterion) have been synthesized and tested against bacteria. Generally, these complexes showed moderate to high activity on Gram-positive bacteria, but were inactive against Gram-negative strains. X-03 (Figure 4), for instance, was active against several Gram-positive bacteria, *S. pneumoniae*, *Listeria monocytogenes*, and *S. aureus*, but showed no toxicity at the tested concentrations against Gram-negative microorganisms. X-03 appears to interfere with iron acquisition systems in *S. pneumoniae* cells, in a similar manner to R-825. Proteomic data revealed that X-03 caused the downregulation of several proteins involved in oxidative stress response and fatty acid biosynthesis, suggesting a mechanism of action based on increased susceptibility to oxidative stress and membrane damage. Additionally, X-03 displayed low toxicity even at a concentration 8 times higher than the MIC value to the A549 alveolar and HBE bronchial epithelial cell lines, indicating selective toxicity against bacteria [42].

Complexes with photolabile ligands, in which L is unidentately coordinated, L = 4-(4-chlorobenzoyl)pyridine (clbzpy), Y = PF_6_‾, *n* = 1 ([Ru(bpy)_2_Cl(clbzpy)]^+^, Figure 4), was moderately active against *S. aureus* and *S. epidermidis*. Additionally, the complex was shown to suffer blue light photolysis (453 nm) in aqueous solution and the resulting photoproduct, *cis*-[Ru(bpy)_2_(H_2_O)Cl]^+^, displayed high binding affinity towards DNA in vitro. The antibacterial activity, however, was not influenced by blue light irradiation, which indicates that the antibacterial activity is not due to DNA damage, but might be the result of bacterial membrane disruption [43]. Blue LED irradiation, however, has been shown to enhance the activity of [Ru(bpy)_2_(methionine)]^2+^, albeit not drastically, against *S. aureus* and *S. epidermidis* [44]. Methionine release and subsequent exchange with water molecules via photolysis at 453 and 505 nm in aqueous solution lead to *cis*-[Ru(bpy)_2_(H_2_O)_2_]^2+^, which can bind covalently to double-stranded DNA [44,82] and promote photocleavage [44].

[Ru(bpy)_2_L]Y_n_ complexes, where L = BTPIP, ETPIP, CAPIP, Y = ClO_4_‾, *n* = 2, [Ru(dmb)_2_(ETPIP)]^2+^, and [Ru(phen)_2_(ETPIP)]^2+^ (see Figure 4 for the chemical structures and the IUPAC names of the ligands) displayed good activities against drug-susceptible *S. aureus*. [Ru(bpy)_2_(BTPIP)]^2+^ was the most active compound of the series (MIC = 0.016 mg/mL) and was shown to inhibit biofilm formation and, thus, prevent bacteria from developing drug resistance. [Ru(bpy)_2_(BTPIP)]^2+^ [46] and [Ru(phen)_2_(ETPIP)]^2+^ [45] increased the susceptibility of *S. aureus* to certain aminoglycosidic antibiotics (kanamycin and gentamicin). [Ru(phen)_2_(ETPIP)]^2+^ was found to suppress the gene regulatory activity of the catabolite control protein A (CcpA) in *S. aureus*, which can explain the synergistic effects observed for this complex and kanamycin [45]. Studies conducted on a murine skin infection model for Ru(bpy)_2_(BTPIP)]^2+^ showed that Ru(bpy)_2_(BTPIP)]^2+^ ointments were effective as topical products against skin infection [46]. These complexes, however, have proven to be cytotoxic to A549 cancer cell lines, with IC_50_ values lower than those required for the antibacterial activity [83,84,85,86], which might indicate poor selectivity towards bacteria. To the extent of our knowledge, no cytotoxic tests on normal cell lines have been performed.

**Figure 5:**
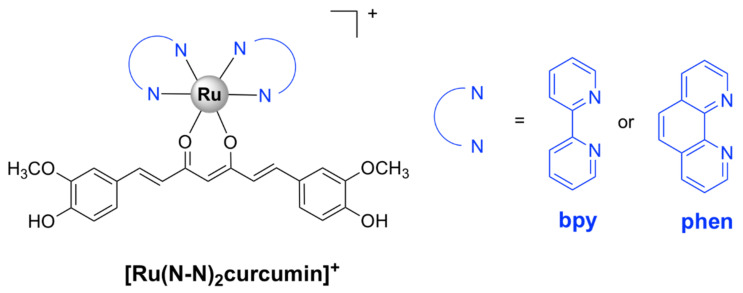
[Ru(N-N)_2_curcumin]^+^, where N-N is either 2,2’-bypiridine (bpy) or 1,10-phenanthroline (phen).

The corresponding ruthenium(II) bipyridine complex in which L = curcumin and Y = PF_6_‾ (Figure 5) was tested against various ESKAPE pathogens. It displayed bactericidal activity against methicillin and vancomycin-resistant *S. aureus* strains (MIC = 1 µg/mL) and high selectivity towards bacteria as compared with eukaryotic Vero cells (SI > 80). Moreover, the complex strongly inhibited biofilm formation in *S. aureus* cells and displayed in vivo antibacterial activity against *S. aureus* comparable to that of vancomycin in a murine neutropenic thigh infection model. However, [Ru(bpy)_2_curcumin]^+^ was not toxic to the Gram-negative *E. coli*, *K. pneumoniae*, *A. baumanii*, and *P. aeruginosa* cells. In comparison, the corresponding Ru(II) complex, [Ru(phen)_2_curcumin]^+^, bearing 1,10-phenanthroline (Figure 5), was also active against the Gram-negative *A. baumanii* with a MIC value comparable to that of levofloxacin, in addition to its activity on the Gram-positive *S. aureus* bacteria and lack of toxicity against eukaryotic cells [47].

##### Mononuclear Ru(II) Heteroleptic Complexes Bearing 1,10-phenanthroline (phen)

**Figure 6:**
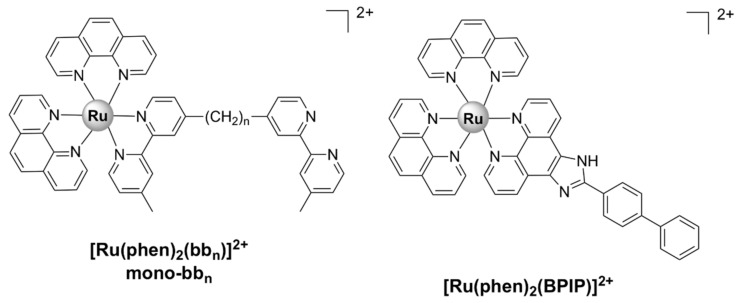
Chemical structures of heteroleptic Ru(II) complexes bearing 1,10-phenanthroline (phen) ligands.

Mononuclear Ru(II) complexes bearing phenanthroline ligands have also been investigated as potential antibacterial agents. Amongst these complexes, mono-bb_n_ ([Ru(phen)_2_bb_n_]^2+^) (Figure 6), where bb_n_ is bis[4(4’-methyl-2,2’-bipyridyl)]-1,*n*-alkane and *n* stands for the number of methylene groups in the alkane chain of bb_n_ (*n* = 7 or 10), have been extensively investigated. Although mono-bb_10_ has a larger alkane chain and therefore is more lipophilic, it was less active than mono-bb_7_ against drug-susceptible *S. aureus* [38,87,88]. The bactericidal activity of mono-bb_7_ was linked to the extent of cellular accumulation, since its activity on Gram-negative strains is low and the uptake in *Staphylococcus* strains is much higher than in *E. coli* or *P. aeruginosa* [37,38]. Mono-bb_7_ caused membrane depolarization in *S. aureus* cells and increased membrane permeability, which might suggest the membrane damage as part of its mode of action [88]. Morphological changes indicative of membrane damage have also been reported for a similar complex, [Ru(phen)_2_(BPIP)]^2+^, where BPIP = 2-(4′-biphenyl)imidazo[4,5-*f*][1,10]phenanthroline (Figure 6), in Gram-positive (*Micrococcus tetragenus* and *S. aureus*) bacteria [76]. Mono-bb_7_ displayed selective activity against bacterial over healthy mammalian cells [38,89].

**Figure 7:**
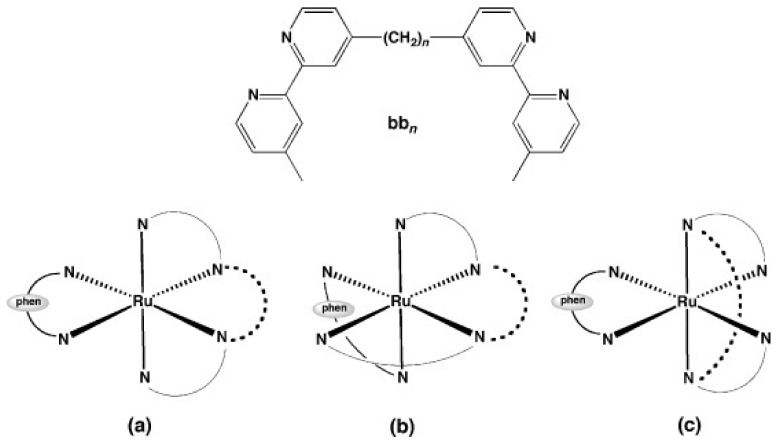
The ligand bb_n_ and the possible isomeric forms of the mononuclear complex [Ru(phen)(bb_n_)]^2+^ with bb_n_ as a tetradentate ligand: (**a**) *cis*-α isomer, (**b**) *cis*-β isomer, and (**c**) a form in which the central polymethylene chain spans the *trans*. Reproduced from [48] with permission. Copyright © 2015 WILEY-VCH Verlag GmbH & Co. KGaA, Weinheim.

A complex in which the bb_12_ ligand is tetradentately bound to Ru (II), *cis*-α-[Ru(phen)bb_12_]^2+^ (Figure 7a, see for comparison the other isomers of the compound, depicted in Figure 7b,c), was found to be more active against the Gram-negative *P. aeruginosa* than the more lipophilic mono-bb_7_. The activity was found to be positively correlated with the uptake of the complex into the cells. Nonetheless, *cis*-α-[Ru(phen)bb_12_]^2+^ was still considerably more active against Gram-positive bacteria as compared with *P. aeruginosa*, the compound being more active against MRSA than ampicillin and gentamicin. Interestingly, *cis*-α-[Ru(phen)(bb_12_)]^2+^ was found to be two to four times more active than its geometric isomer, *cis*-β-[Ru(phen)(bb_12_)]^2+^, against the Gram-negative strains (*E. coli* and *P. aeruginosa*), while no difference in activity was found for the Gram-positive bacteria (*S. aureus* and MRSA). It is unclear why the *cis*-α isomer is more active, since no significant difference in cellular accumulation was observed for the two isomers. Moreover, both geometric isomers were shown to bind tightly and with similar potency to duplex DNA in vitro, but no correlation between the binding constants and activity was found [48]. It should be noted that DNA/RNA binding is a possible mechanism of action for these complexes, since several reports indicate that various inert Ru(II) polypyridyl complexes bearing phenanthroline ligands target DNA and RNA in bacterial and eukaryotic cells [76,90,91]. Notably, the similar complex *cis*-α-[Ru(Me_4_phen)(bb_7_)]^2+^ displayed similar activity towards Gram-positive and Gram-negative bacteria as *cis*-α-[Ru(phen)(bb_12_)]^2+^ and remarkably high DNA binding affinity (~10^7^) [92].

##### Mononuclear Ru (II) Heteroleptic Complexes Bearing Pyridophenazine Ligands

**Figure 8:**
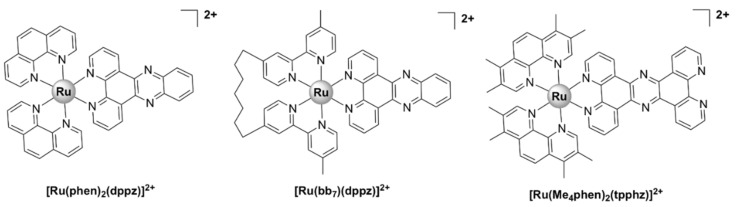
Chemical structures of heteroleptic Ru (II) complexes bearing pyridophenazine ligands.

[Ru(phen)_2_(dppz)]^2+^ (Figure 8), where dppz = dipyrido[3,2-*a*:2’,3’-c]phenazine and phen = 1,10-phenanthroline, displayed good bactericidal activity against *M. smegmatis* (MIC = 2 µg/mL). Its mechanism of action was suggested to be linked to ROS generation and DNA intercalation [93]. A similar complex, [Ru(2,9-Me_2_phen)_2_(dppz)]^2+^, was active against MRSA and *B. subtilis*, and displayed time–kill curves that were similar to those of currently used antibiotics, but displayed no activity against *E. coli*. The activity appeared to be correlated with the ability to intercalate into DNA double strands in vitro. In vivo antibacterial activity has been assessed using the nematode *Caenorhabditis elegans* infection model and [Ru(2,9-Me_2_phen)_2_(dppz)]^2+^ proved to be non-toxic to the nematodes [40].

[Ru(bb_7_)(dppz)]^2+^ (Figure 8) (bb_7_ = bis[4(4’-methyl-2,2’-bipyridyl)]-1,7-alkane) was 2–8 fold more active than its parent compound [Ru(phen)_2_(dppz)]^2+^ against both Gram-positive (*S. aureus*, MRSA) and Gram-negative bacteria (*E. coli*, *P. aeruginosa*). Although the two complexes have comparable lipophilicity, [Ru(bb_7_)(dppz)]^2+^ accumulated in *P. aeruginosa* to the same degree as in MRSA and was shown to permeabilize a model membrane system to a higher degree than [Ru(phen)_2_(dppz)]^2+^. Therefore, its higher cellular uptake might be responsible for the increase in activity. However, Ru(bb_7_)(dppz)]^2+^ was also ~3-fold more toxic to healthy eukaryotic cells than [Ru(phen)_2_(dppz)]^2+^, while still being more active against bacterial cells [49].

Complexes bearing tetrapyridophenazine (tpphz) are more lipophilic relative to their dppz analogues and generally more active. For instance, the luminescent, mononuclear ruthenium(II) complex bearing the tpphz ligand, [Ru(Me_4_phen)_2_(tpphz)]^2+^ (Figure 8), displayed a comparable activity to that of ampicillin and oxacillin in drug-sensitive strains and the activity was retained in resistant strains. The complex was taken up by both Gram-positive (*E. faecalis*, *S. aureus*) and Gram-negative (*E. coli*, *A. baumannii*, *P. aeruginosa*) bacteria in a glucose-independent manner and was shown to target chromosomal DNA in both Gram-positive and Gram-negative strains. Moreover, model toxicity screens showed that the compound is non-toxic to *Galleria mellonella* larvae at concentrations that are 3–25 times higher than the MIC values [50]. This complex represents the starting point for the kinetically inert dinuclear polypyridylruthenium(II) complex [Ru_2_(Me_4_phen)_2_(tpphz)]^4+^ (see below), which displayed higher antibacterial activity (Table 1), except against *S. aureus*. Unlike the dinuclear derivative, [Ru(Me_4_phen)_2_(tpphz)]^2+^ does not cause membrane damage.

#### 5.1.2. Mononuclear Ru (II)–arene Complexes

**Figure 9:**
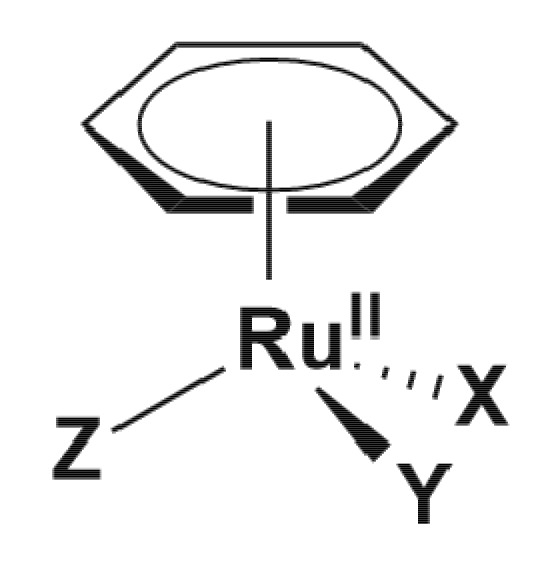
Representative ‘piano stool’ Ru^II^-η^6^–arene complex, where X, Y, and/or Z is a labile ligand.

Due to the promising anticancer activities of some representatives, the potential antibacterial properties of piano-stool Ru(II)-η^6^–arene complexes, with the general structure shown in Figure 9, have also been considered for antimicrobial applications [94,95,96,97,98,99,100,101,102,103]. While some of them displayed modest activity [76,79,80], complexes of the general formulae [Ru(η^6^-*p*-cymene)X_2_(PTA)] (RAPTA-C complexes), where X = Cl, Br, I, NCS (labile) and PTA = 1, 3, 5-triaza-7-phosphaadamantane, were active in different degrees against bacteria (*E. coli*, *B. subtilis*, *P. aeruginosa*) and fungi (*Candida albicans*, *Cladosporium resinae,* and *Trichrophyton mentagrophytes*). The PTA ligand was suggested to play a role in facilitating the uptake of the complex into bacterial cells, while the antimicrobial activity was suggested to be mediated by the interaction of the Ru(II) ion with intracellular proteins. Although the complexes were found to cause DNA damage in vitro, their affinity towards DNA was not correlated with their antibacterial activities. Interestingly, extracts from *E. coli* cells treated with a PTA derivative show specific protein–ruthenium interactions, suggesting that the intracellular proteins are most likely targets of these complexes [94].

**Figure 10:**
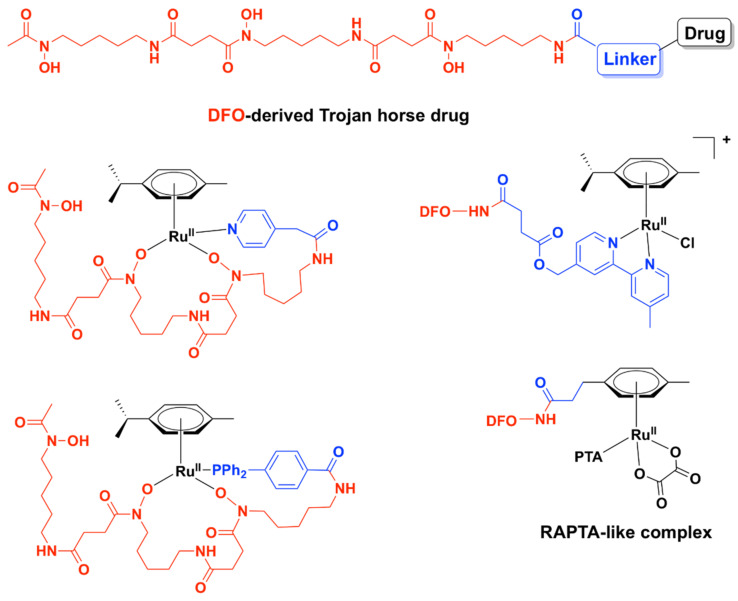
General structure of deferoxamine B (DFO)-derived Trojan Horse antibacterial drugs and some DFO-derived Ruthenium(II)–Arene Complexes [104].

Relying on potential interference with the iron-acquisition systems and in order to increase internalization of the complexes in bacteria, a Trojan Horse strategy was applied for three Ru (II)–arene complexes and one RAPTA-like complex bearing derivatives of deferoxamine B (DFO) (Figure 10) [104]. DFO is a commercially available siderophore, namely an iron chelator that is secreted by microorganisms to bind extracellular Fe (III) and aid in its transport across bacterial membranes inside the cells [105]. These compounds displayed only modest activity against three ESKAPE pathogens (*S. aureus*, *K. pneumoniae*, *A. baumannii*) and one fungal strain (*C. albicans*) when Fe (III) ions were present in the medium. Absence of iron in the media led to an increase in activity, particularly for *K. pneumoniae*. All Ru (II) complexes of this series, however, showed little to no activity against *P. aeruginosa*, *E. coli*, and *C. neoformans*, presumably because these bacterial and fungal strains are more susceptible to internalizing DFO. Antiproliferative studies on normal cells (HEK-293) showed that these complexes were essentially non-toxic towards normal eukaryotic cells in the presence of iron [104].

Various Ru(II)–arene complexes with thiosemicarbazone ligands were more active against Gram-positive bacteria than Gram-negative bacteria and/or fungi, but were still less active than the antibiotics used as controls (ampicillin, streptomycin, or ciprofloxacin) [95,98,100,106]. As was seen for other ruthenium complexes, they were shown to bind DNA and human serum albumin with significant affinity in vitro, suggesting that DNA and/or proteins are potential targets of these complexes in bacterial cells. Several complexes were shown to exert low cytotoxicity towards healthy cell lines [95].

Ru(II)-η^6^-*p*-cymene complexes bearing pyrazole derivatives containing *N,S* donor atoms exerted moderate antibacterial activity against Gram-positive strains, including *S. aureus*, *S. epidermidis*, and *E. faecalis*, while displaying very weak to no activity against Gram-negative bacteria (*P. vulgaris*, *P. aeruginosa*). Notably, the complexes were non-toxic against the healthy human fibroblast HFF-1 cells [107]. Other Ru(II)–arene complexes with various *N,N*- or *N,O*- bidendate ligands displayed moderate activity against various Gram-positive bacterial strains and, notably, were found to be more active against *P. aeruginosa* than various clinically used antibiotics used as controls [96,99].

While it is well known that Ru(II)–arene complexes have been widely investigated as potential anticancer agents, their clinical use as antibacterial drugs may be limited by their cytotoxic effects (and generally the poor selectivity for cancerous over healthy cells). Some of these complexes, however, exhibited dual antibacterial and anticancer activities [104]. This constitutes a desirable trait as current anticancer therapy weakens the immune system and often leaves patients susceptible to opportunistic infections. Conversely, patients suffering from a chronic infection are more prone to develop cancer due to certain defects in the immune response [108].

#### 5.1.3. Other Mononuclear Ru Complexes

Various other Ru(II/III) complexes have been reported to possess antibacterial activity. However, microbiological studies for these complexes mainly involved disc diffusion assays or MIC testing, without any further research with regard to their modes of action [109,110,111,112,113,114,115,116,117,118,119,120]. These complexes were generally more active against Gram-positive strains, with little to no activity against Gram-negative or drug-resistant bacteria. However, a Ru(III) complex, [Ru(L)Cl_2_]Cl, where L is a N,N,N,N- tetradentate macrocyclic ligand derived from 2,6-diaminopyridine and 3-ethyl-2,4-pentanedione, was moderately active against the Gram-negative bacteria *Xanthomonas campestris* and *P. aeruginosa* and displayed higher activity than the corresponding Pd(II), Pt(II), and Ir(III) complexes [114]. Three ruthenium half-sandwich complexes containing phenyl hydrazone Schiff base ligands also displayed good activity against the Gram-negative *P. aeruginosa*, comparable to that of the positive control, gentamicin, and generally higher than the corresponding Ir(III) and Rh(III) complexes [111].

**Figure 11:**
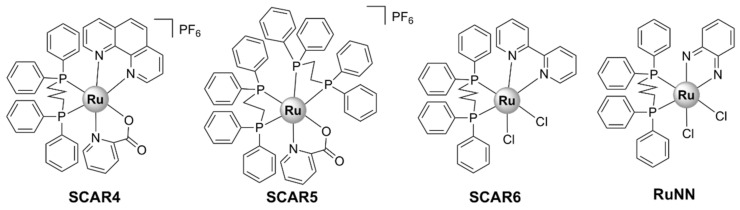
Chemical structures of selected SCAR complexes and RuNN.

There are few examples of Ru(II) complexes that display antimycobacterial activity. However, ‘SCAR’ compounds, consisting of a series of Ru(II) complexes containing phosphine/picolinate/diimine ligands (Figure 11), had low MIC values against multidrug-resistant strains of *M. tuberculosis* [51,121,122]. Moreover, the SCAR complexes exerted synergistic interactions with first-line antibiotics, with the best overall synergistic activity observed with isoniazid [122]. Although these complexes displayed some selectivity towards bacterial over healthy eukaryotic cells, an increase in the toxic effects against bacteria was correlated with higher toxicity against eukaryotic cells. *Cis*-[RuCl_2_(dppb)(bpy)] (SCAR6), where dppb = 1,4-bis(diphenylphosphino)butane and bpy = 2,2’-bipyridine, the least active compound of the series, was found to be the least stable in aqueous solutions [121]. Upon dissolution in water, the chlorido ligands are released, and the resulting species was shown to bind covalently to DNA and induce DNA damage in a similar manner to cisplatin [51,121]. Moreover, the metabolic products of SCAR6 were responsible for the mutagenic effects of the compound observed in *Salmonella typhimurium*. In contrast, SCAR4 and SCAR5 did not display any mutagenic effect [51].

A biphosphinic ruthenium complex, *cis*-[Ru(dppb)(bqdi)Cl_2_]^2+^ (Figure 11, RuNN), where dppb = 1,4-bis(diphenylphosphino)butane and bqdi = o-benzoquinonediimine, displayed bacteriostatic and bactericidal activity against Gram-positive bacteria (*S. aureus*, including MRSA, and *S. epidermidis*). Time–kill kinetics studies indicated that RuNN displayed bactericidal activity in the first 1–5 h [52]. Note that this is a much shorter time than that reported for vancomycin or telavancin (24 h) [123]. Additionally, the combination treatment of RuNN and ampicillin (but not tetracycline) resulted in a dramatic increase in activity, highlighting the synergistic effect of the two drugs against *Staphylococcus* spp. For the drug-resistant *S. epidermidis* ATCC 35,984 strain, the MIC value for the RuNN + ampicillin treatment was 1/16 of that of ampicillin alone. Furthermore, RuNN inhibited the formation of *S. aureus* biofilms and reduced the total biomass of mature biofilms by ~50%. The complex displayed no hemolytic activity on erythrocytes [52].

Several ruthenium complexes with antibiotics have been reported. The activity of trimethoprim was, unfortunately, significantly decreased upon complexation with Ru(III) [124]. Complexes of the half-sandwich Ru(II)–arene complex [Ru(η^6^-*p*-cymene)] with a ciprofloxacin derivative, CipA, exhibited higher activity against *E. coli* and *S. aureus* than CipA. These complexes are labile in aqueous solutions and, therefore, their activity is probably the result of additive or synergistic effects of the [Ru(η^6^-*p*-cymene)] complex and CipA [125]. Ru(II) complexes with clotrimazole were active against mycobacteria, but were also found to be significantly toxic to mammalian cells [126]. Three Ru(III) complexes of ofloxacin, namely [Ru(OFL)_2_(Cl)_2_]Cl [Ru(OFL)(AA)(H_2_O)_2_]Cl_2_, where OFL = ofloxacin and AA is either glycine or alanine, were active against Gram-negative bacteria (*E. coli* and *K. pneumoniae*), but showed little to no activity on Gram-positive bacteria (*S. epidermidis*, *S. aureus*) [127]. This is unsurprising, given that fluoroquinolones are particularly effective against Gram-negative microorganisms [128].

**Figure 12:**
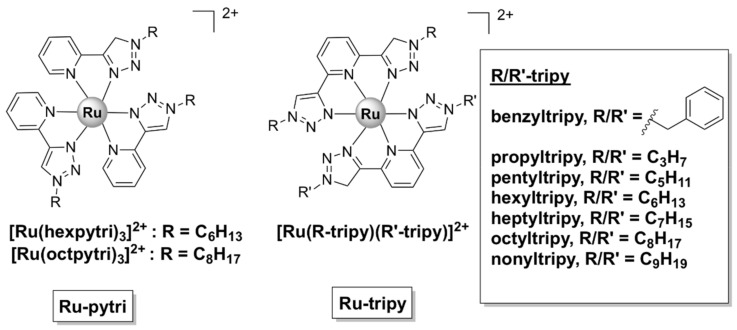
Chemical structures of ruthenium(II) complexes with ‘‘click’’ pyridyl-1,2,3-triazole ligands with various aliphatic and aromatic substituents (generally denoted as Ru-pytri [53] and Ru-tripy [54]). Adapted with permission from [53], Copyright © 2016, American Chemical Society and [54], © 2019 Wiley-VCH Verlag GmbH & Co. KGaA, Weinheim.

Homo- and hetero-leptic ruthenium(II) complexes with ‘‘click’’ pyridyl-1,2,3-triazole ligands with various aliphatic and aromatic substituents (generally denoted as Ru-pytri and Ru-tripy, Figure 12) have been reported to possess good antibacterial activity. Generally, the most active complexes displayed high activity against Gram-positive strains, including MRSA (MIC = 1−8 µg/mL), but were less effective against Gram-negative bacteria (MIC = 8−128 µg/mL) [53,54]. The Ru-tripy series was generally more effective against Gram-negative bacteria than the Ru-pytri compounds [54]. Notably, the water-soluble chloride salts of the most active Ru-pytri complexes ([Ru(hexpytri)_3_]^2+^ and Ru(octpytri)_3_]^2+^, Figure 12) displayed higher activity than the gentamicin control against two strains of MRSA (MR 4393 and MR 4549). Moreover, the Ru-pytri complexes exhibited only modest cytotoxic effects at concentrations higher than the MIC values on Vero (African green monkey kidney epithelial) and human dermal keratinocyte cell lines [53]. For the Ru-tripy series, the activity appears to be closely linked to the length of the alkyl chain, with hexyl or heptyl substituents on the “click” ligands resulting in the highest activity of the corresponding homo- and hetero- leptic Ru(II) complexes. The MIC values for the most active complex of the Ru-tripy series, [Ru(hexyltripy)(heptyltripy)]Cl_2_, were 2 μg/mL and 8 μg/mL, respectively, against *S. aureus* and *E. coli*. Despite being generally more active than the Ru-pytri series, the Ru-tripy complexes demonstrated little to no selectivity for prokaryotic vs. eukaryotic cells (IC_50_ = 2–25 µM on eukaryotic cells lines—cancer and skin). With regard to their mechanism of action, transmission electron microscopy (TEM) experiments and propidium iodide assays identified cell wall/cytoplasmic membrane disruption as the main mechanism for the Ru-pytri complexes [53], while [Ru(hexyltripy)(heptyltripy)]Cl_2_ appears to cause abnormal cellular division [54].

Chitosan Schiff base derivatives conjugated to Ru(III) ions give polymers enhanced water solubility and antibacterial activity against Gram-positive (*B. subtilis* and *S. aureus)* and Gram-negative (*E. coli*, *K. pneumoniae,* and *P. aeruginosa*) bacteria [79].

### 5.2. Polynuclear Ruthenium (II) Complexes

#### 5.2.1. Kinetically Inert Dinuclear Polypyridylruthenium (II) Complexes

**Figure 13:**
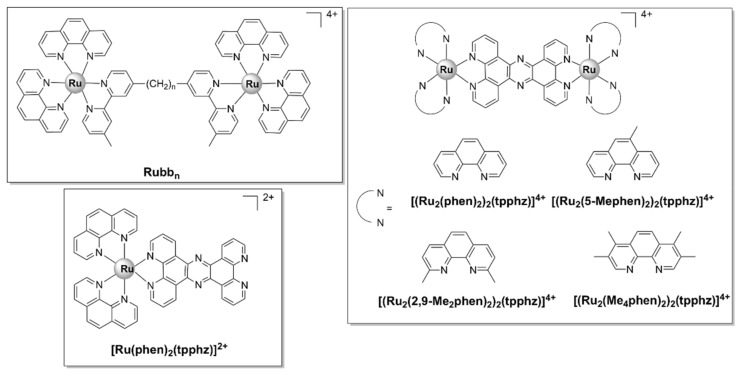
Chemical structures of the inert dinuclear Rubb_n_ ([Ru_2_(phen)_2_(tpphz)]^4+^, [Ru_2_(5-Mephen)_2_(tpphz)]^4+^, [Ru_2_(2,9-Me_2_phen)_2_(tpphz)]^4+^, and [Ru_2_(Me_4_phen)_2_(tpphz)]^4+^) and mononuclear ([Ru(phen)_2_(tpphz)]^2+^) complexes.

The ruthenium polynuclear complexes, commonly known as Rubb_n_, are the most investigated ruthenium-based compounds with regard to their antimicrobial activities. Rubb_n_ are kinetically inert dinuclear polypyridylruthenium (II) complexes with the general formula [(Ru(phen)_2_)_2_(μ-bb_n_)]^4+^ (Figure 13), where bb_n_ = bis[4(4’-methyl-2-2’-bipyridyl)]-1, *n*-alkane. In the dinuclear Rubb_n_ complexes, two mononuclear mono-bb_n_ fragments (described above) are bridged by a flexible methylene linker, bb_n_, where *n* represents the number of methylene groups in the alkyl chain. Rubb_n_ are moderately active against Gram-negative bacteria (*E. coli*, *P. aeruginosa*) and exhibit excellent activity against Gram-positive strains (including MRSA—MIC Rubb_12/16_ = 1 mg/L, while MIC gentamicin = 16 mg/L) [37,38]. The antibacterial activity appears to be closely linked to cellular uptake, which was, in turn, shown to be directly proportional to the length of the alkyl chain and therefore the lipophilicity of the compounds [38]. Of note, a follow-up study comparing the mononuclear [Ru(Me_4_phen)_3_]^2+^ (Figure 3) with the dinuclear Rubb_n_ complexes reported significant differences in the cellular uptake and mode of action. While Rubb_n_ are taken up by *S. aureus* cells via a passive transport mechanism, the cellular uptake of [Ru(Me_4_phen)_3_]^2+^ appears to be protein-mediated (active transport) [88]. In eukaryotic cells, however, Rubb_n_ complexes are transported via either an active or a passive mechanism depending on the cell type and have been shown to localize to the mitochondria or the RNA-rich nucleolus [56,91,129].

The large positive charge (+4) and the hydrophobic alkyl chain are key structural features that contribute to the activity of the Rubb_n_ complexes, allowing these compounds to pierce the bacterial cell walls and exert antibacterial activity. Based on the knowledge gained so far, two modes of action have been reported for dinuclear Rubb_n_ complexes: membrane damage and/or interaction with nucleic acids, specifically ribosomal RNA.

Rubb_n_ complexes were found to depolarize and permeabilize the membranes of *S. aureus* cells, while no membrane permeabilization was observed for [Ru(Me_4_phen)_3_]^2+^, although it did cause depolarization [88]. Additionally, Rubb_12_ was shown to embed via a pore-formation mechanism into negatively charged phospholipid multilamellar vesicles, an artificial model generally used to study drug–membrane interactions in vitro [130]. Interestingly, the corresponding Ir(III) complex, Irbb_12_ (with a formal charge of +6), was not taken up by cells and was inactive [60]. Molecular dynamics (MD) simulations showed that the bulky, positively charged Rubb_12_ spanned the bacterial membrane model at the negatively charged glycerol backbone and the bb_12_ linker threaded the hydrophobic core. It is yet to be determined whether the interaction with bacterial membranes results in a change of state (fluidity, charge) of the membrane and if it plays a part in the activity of Rubb_12_. It should be noted that the complex only interacted at the surface level with a neutrally charged eukaryotic membrane model, which could explain its lower toxicity towards healthy cells vs. bacteria (see below) [130]. This does not exclude the possibility of a protein-mediated transport of Rubb_12_ inside eukaryotic cells.

The bactericidal mechanism of these complexes [38] was originally presumed to be linked to their ability to bind DNA [131,132]. Indeed, the dinuclear polypyridyl complex [(phen)_2_Ru-(μ-tpphz)-Ru(phen)_2_]^4+^ [133] and Rubb_7_ [132] were found to localize to *S. aureus* chromosomal DNA. However, despite binding with reasonably high affinity to double-stranded DNA in vitro, Rubb_n_ complexes prefer non-duplex structures such as bulges and hairpins[132,134,135]. Live cell microscopy experiments on *E. coli* cells showed that Rubb_16_ was found to localize at polysomes, with negligible binding to chromosomal DNA. Polysomes are formed when multiple ribosomes associate along the coding region of mRNA and therefore play an essential role in protein synthesis. The cationic charge of Rubb_16_ is thought to promote its interaction with the highly negatively charged polysomes. Furthermore, Rubb_16_ was found to induce condensation of the polysomes, an effect which is thought to hinder protein production and therefore inhibit bacterial growth [90]. Rubb_n_ also displayed high affinity towards the serum transport proteins albumin and transferrin in vitro, which suggests that these complexes could potentially target intracellular proteins [88].

As was shown for Rubb_16_, targeting ribosomal RNA (rRNA) in bacteria can be advantageous for the development of selective antibacterial agents, since there are significant differences between prokaryotic and eukaryotic rRNA [136]. Moreover, in vitro experiments and MD simulations have shown that Rubb_12_ only interacts at a surface level with a neutral membrane bilayer mimic of a eukaryotic membrane [130]. Indeed, these inert Ru(II) complexes generally display selectivity for bacteria over normal eukaryotic cells. Although toxic to cancer cells, Rubb_12/16_ were much less active (up to 100-fold) against healthy cell lines [89,90,129]. In spite of the fact that Rubb_16_ is slightly more active against bacteria than Rubb_12_, the higher in vitro toxicity of Rubb_16_ to both healthy eukaryotic cells and red blood cells makes Rubb_12_ a more promising drug candidate [37].

Rubb_12_ injected intramuscularly was not toxic to mice at concentrations up to 64 mg/kg. Moreover, pharmacokinetic experiments have shown that 30 min post-administration, serum concentrations of Rubb_12_ are higher than the MIC values for Gram-positive bacteria and were maintained for more than 3 h [55]. Encapsulation of Rubb_12_ in cucurbit[10]uril (Rubb_12_⊂Q[10]) resulted in a two-fold decrease in toxicity (free Rubb_12_—1 mg/kg, Rubb_12_⊂Q[10]—2 mg/kg) when administered intravenously to mice. Interestingly, while free Rubb_12_ accumulated predominantly in the liver, Rubb_12_⊂Q[10] was found to be distributed in comparable amounts in both the liver and kidneys. A substantial reduction (∼2-fold) in the ruthenium concentrations (quantified using Inductively Coupled Plasma Mass Spectrum, ICP-MS) found in the liver was reflected by an increase (∼4-fold) in the kidneys. The significant increase in kidney accumulation is the result of the renal excretion of Rubb_12_⊂Q[10]. The encapsulation in cucurbit[10]uril resulted in higher cellular accumulation, lower toxicity, and faster clearance of Rubb_12_ [137].

As opposed to Rubb_n_, which bear flexible linkers, systems bridged by a rigid, extended aromatic ligand possess a property that is rather unusual for this class of complexes, that is a generally higher activity against pathogenic Gram-negative as compared with Gram-positive bacteria. The more rigid structure of these complexes is thought to play an essential role in their activity against Gram-negative strains, as well as the presence of potentially ionizable nitrogen sites and the more complex 3D structure when compared with typical drug architectures [57,138]. Thus, a range of luminescent dinuclear Ru(II) complexes bearing tetrapyridophenazine (tpphz) (Figure 13) were found to be more active against Gram-negative (both a wild-type and a multidrug-resistant strain of *E. coli*) than Gram-positive (a vancomycin resistant strain of *E. faecalis*) bacteria. [(Ru_2_(5-Mephen)_2_)_2_(tpphz)]^4+^ was the least active compound of the series, most likely due to its low water solubility. For the other three complexes, a direct, positive relationship was observed between lipophilicity and activity. The lead compound of the series, [Ru_2_(Me_4_phen)_2_(tpphz)]^4+^, was also non-toxic to healthy eukaryotic cells (Table 1). Of note, all complexes showed appreciable activity against the ESKAPE pathogens and [Ru_2_(Me_4_phen)_2_(tpphz)]^4+^ even displayed higher activity than ampicillin against the wild-type strain of *E. coli* and against *E. faecalis*. Selectivity towards the Gram-negative strains has also been observed for the mononuclear parent compound, [Ru(phen)_2_(tpphz)]^2+^, even though it was found to be significantly less active than its dinuclear derivatives against all bacterial strains [57].

[Ru_2_(Me_4_phen)_2_(tpphz)]^4+^ was shown to be actively taken up into Gram-negative bacterial cells and to disrupt the bacterial membrane structure before internalization [57], results which were further substantiated by transcriptomic analysis. Thus, the complex caused a significant downregulation of genes involved in membrane transport and the tricarboxylic acid cycle and upregulation of the *spy* gene [58]. The *spy* gene, encoding a periplasmic chaperone, is involved in zinc homeostasis and in maintaining the homeostasis of protein folding under cellular stress [139]. Thus, overexpression of the *spy* gene in the [Ru_2_(Me_4_phen)_2_(tpphz)]^4+^-stressed cells indicates protein damage in the outer membrane. Moreover, multi-drug resistant *E. coli* cells developed resistance to [Ru_2_(Me_4_phen)_2_(tpphz)]^4+^ much slower, and only in low levels, in comparison with various clinically available antibiotics. Encouragingly, [Ru_2_(Me_4_phen)_2_(tpphz)]^4+^ was active at low micromolar concentrations against other Gram-negative ESKAPE pathogens, including *P. aeruginosa* and *A. baumannii* [58].

A similar mode of action involving membrane and DNA damage was reported in the less susceptible, Gram-positive *S. aureus* cells. However, [Ru_2_(Me_4_phen)_2_(tpphz)]^4+^ was found to accumulate to a lower extent in Gram-positive when compared with Gram-negative bacteria, which may account for the lower efficacy of these complexes against the former. This was shown to be related to a resistance mechanism developed by Gram-positive bacteria against cationic species, which involves upregulation of the *mprF* gene. Overexpression of this gene leads to the accumulation of positively charged phospholipids on the outer leaflet of the cytoplasmic membrane, which repel cationic molecules, such as metal complexes. Consequently, it was found that [Ru_2_(Me_4_phen)_2_(tpphz)]^4+^ was more active against a *mprF*-deficient *S. aureus* strain and in mutant *S. aureus* strains missing, or with altered, wall teichoic acids [59].

This class of compounds, particularly [Ru_2_(Me_4_phen)_2_(tpphz)]^4+^, shows remarkable promise for the treatment of infections caused by Gram-negative pathogens. In addition, the lead compound displays good kinetic solubility, which suggests good bioavailability and possible oral administration [58]. Clearly, animal experiments are needed to further assess the efficacy of this class of compounds as novel antibacterial agents in vivo.

#### 5.2.2. Chlorido Dinuclear Polypyridylruthenium (II) Complexes

**Figure 14:**
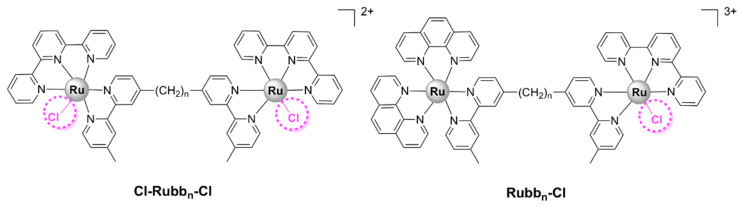
Chemical structures of labile dinuclear Cl-Rubb_n_-Cl and Rubb_n_-Cl complexes, where *n* = 7, 12, 16.

A range of symmetrical dinuclear polypyridylruthenium(II) complexes with the general formula [(Ru(terpy)Cl)_2_(μ-bb_n_)]^2+^ (where terpy = 2,2’:6’,2’’-terpyridine) have been reported [55,60]. These labile complexes are commonly denoted as Cl-Rubb_n_-Cl (Figure 14). These complexes have a positive charge of +2; however, upon dissolution in water followed by the substitution of the chloride ions with solvent molecules, their charge increases to +4 [60]. The Cl-Rubb_7/12/16_-Cl complexes exert bactericidal activity against Gram-positive strains (*S. aureus* and MRSA), *E. coli*, and *P. aeruginosa*, with Cl-Rubb_12_-Cl being the lead compound of the series. Cl-Rubb_7/12_-Cl are more active than their dinuclear inert analogues; however, the Cl-Rubb_16_-Cl complex was significantly less active than Rubb_16_ [60]. It is uncertain why this variation occurs, but a possible reason is speculated to be that the enhanced cellular uptake of the Cl-Rubb_7/12_-Cl complexes can compensate for a reduction in activity. Since Rubb_16_ readily accumulates into cells, the addition of chlorido groups only results in a lower activity.

Asymmetrical chloride-containinig dinuclear polypyridylruthenium(II) complexes, Rubb_7/12/16_-Cl (Figure 14), have also been reported. The Rubb_n_-Cl complexes contain two ruthenium centers bridged by a flexible methylene linker. One ruthenium center bears a labile chlorido ligand, while the second is kinetically inert. The MIC values calculated for these complexes are comparable with those reported for the previously described Cl-Rubb_7/12/16_-Cl series. Furthermore, with the exception of Rubb_16_-Cl, Rubb_n_-Cl complexes exert superior antibacterial activities as compared with their inert dinuclear analogues, with Rubb_12_-Cl being the most active against both Gram-positive and Gram-negative strains. Rubb_12_-Cl was found to be 30- to 80-fold more toxic to the bacteria than to eukaryotic cell lines—two healthy cell lines (baby hamster BHK and embryonic HEK-293 kidney) and one cancerous cell line (liver carcinoma HepG2). Interestingly, large differences were found in the cytotoxic activity of Rubb_7_-Cl as compared with Rubb_12/16_-Cl. It was significantly more active towards the Gram-negative *E. coli* than against the Gram-positive *S. aureus* and MRSA, significantly more toxic to HepG2 (IC_50_ = 3.7 µM), and far less toxic to BHK (IC_50_ = 238 µM) cells than Rubb_12/16_-Cl. Cellular localization studies in HepG2 cells suggest that all complexes of this series were shown to accumulate preferentially in the rRNA-rich nucleolus. In addition, the large differences in the toxicity profile of the Rubb_n_-Cl complexes might be related to the fact that Rubb_7_-Cl binds to chromosomal DNA to a greater extent than Rubb_12/16_-Cl [56].

#### 5.2.3. Tri-/Tetra-Nuclear Polypyridylruthenium(II) Complexes

**Figure 15:**
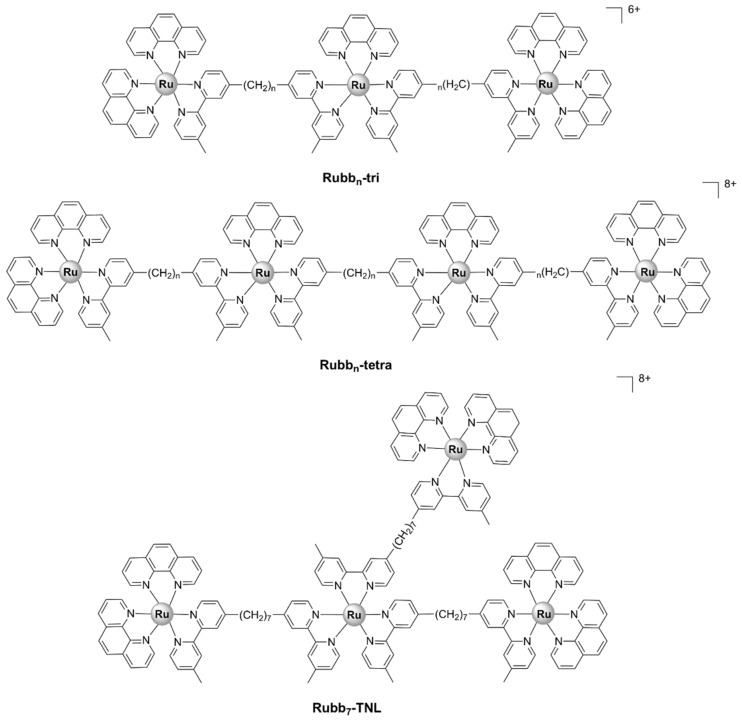
Chemical structures of inert tri- and tetra- nuclear ruthenium complexes, where *n* = 7, 10, 12 or 16.

Generally, the more lipohilic tri- and tetra- nuclear complexes, Rubb_7/10/12/16_-tri and Rubb_7/10/12/16_-tetra (Figure 15), displayed higher activities against Gram-positive and Gram-negative strains, as well as a range of bacterial clinical isolates, than the dinuclear Rubb_n_ complexes [37,61]. The linear tetranuclear [Rubb_n_-tetra]^8+^ complexes were more active, with MIC values < 1 µM against Gram-positive bacteria, than their non-linear trinuclear [Rubb_n_-tri]^6+^ analogues. Time–kill curve experiments showed that Rubb_12_-tri and Rubb_12_-tetra exert bactericidal activity and kill bacteria within 3–8 h [55].

As opposed to the inert dinuclear Rubb_n_ complexes, there is no apparent relationship between the antibacterial activities of the Rubb_n_-tri and Rubb_n_-tetra complexes and lipophilicity or cellular uptake. The more active tetranuclear [Rubb_n_-tetra]^8+^ complexes are less lipophilic than their trinuclear [Rubb_n_-tri]^6+^ analogues, despite the additional non-polar bb_n_ ligand. This is unsurprising, considering the difference in the overall charge of the tri- and tetra- nuclear complexes. Moreover, even though Rubb_n_-tri and Rubb_n_-tetra complexes were more active against Gram-positive bacteria, they were shown to accumulate to a greater extent in Gram-negative bacteria [61]. Within eukaryotic Hep-G2 cells, Rubb_12_-tri and Rubb_12_-tetra have been shown to accumulate preferentially in the RNA-rich nucleolus, as was previously described for the dinuclear Rubb_n_ complexes [91].

The mechanism of action of the Rubb_n_-tri and Rubb_n_-tetra complexes is yet to be determined, but it is thought to be related to their abilities to bind to nucleic acids and/or proteins [1,61]. The Rubb_n_-tri and Rubb_n_-tetra complexes have been shown to interact with single-stranded oligonucletides and proteins in vitro, with significantly higher affinities than their dinuclear analogues [87,88,91]. The mechanism underlying their interactions with the DNA backbone may differ for the linear tetranuclear and the three dimensional non-linear trinuclear species [87].

Two inert polypyridylruthenium(II) tetranuclear complexes, containing the bridging ligand bis[4(4’-methyl-2,2’-bipyridyl)]-1,7-heptane, with linear (Rubb_7_-tetra or Rubb_7_-TL) and non-linear (Rubb_7_-TNL) (Figure 15) structures, displayed good antibacterial activity against both Gram-positive (*S. aureus*, MRSA) and Gram-negative (*E. coli*, *P. aeruginosa*) bacteria. The non-linear (branched) species displayed slightly higher activity than the corresponding linear analogue and accumulated in the nucleolus and cytoplasm but not in the mitochondria [62].

Rubb_n_-tri and Rubb_n_-tetra complexes were found to be more toxic than Rubb_n_ to carcinoma and healthy mammalian cell lines in vitro, with IC_50_ values lower than or comparable to that of cisplatin [55,89,91]. However, the tri- and tetra- nuclear complexes were still ~50-fold more toxic to Gram-positive bacteria and 25 times more toxic to the susceptible Gram-negative strains than to eukaryotic cells [55]. Rubb_7_-TNL was slightly less toxic to healthy eukaryotic BHK cells than its linear analogue (Table 1), yet still more toxic than cisplatin [62]. In comparison, the dinuclear ΔΔ-Rubb_12_ complex was ~100-fold more toxic to Gram-positive bacteria. The cytotoxic effects of the tri- and tetra- nuclear ruthenium complexes towards eukaryotic cells suggest that merely increasing the lipophilicity and charge is likely to result in decreased selectivity. Therefore, the general goal now is the development of new ruthenium complexes that are highly selective towards bacteria.

#### 5.2.4. Other Polynuclear Complexes

The dinuclear [Ru_2_L_3_]^4+^ ruthenium(II) triply stranded helicate, bearing bidentate ‘‘click’’ pyridyl-1,2,3-triazole ligands, displayed modest antimicrobial activity (MIC > 256 µg/mL) [140] as compared with similar mononuclear Ru(II) complexes bearing ‘‘click’’ ligands [53,54]. However, in contrast to the similarly structured [Fe_2_L_3_]^4+^ helicates, the more kinetically inert [Ru_2_L_3_]^4+^ system proved stable over a period of at least 3 days in DMSO solutions [140].

The binuclear ruthenium (III) complexes [RuX_3_L]_2_ (X = Cl, X = Br), [RuX_3_L_1.5_]_2_ (X = Br), and [RuX_3_L_2_]_2_ (X = Br), where L stands for 2-substituted benzimidazole derivatives, were moderately active against Gram-negative bacteria (*E. coli* and *S. typhi*) as tested by the agar diffusion method. The activity on the Gram-positive bacteria *S. aureus* and *Bacillus aureus* was, however, low when compared with the standard antibiotics ampicillin and fluconazole [141].

### 5.3. Hetero-bi/tri-Metallic Complexes

**Figure 16:**
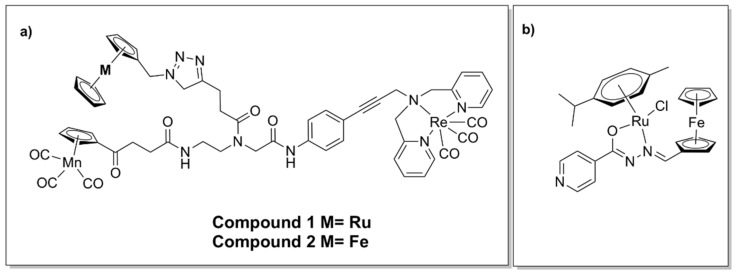
Chemical structures of hetero- (**a**) trimetallic complexes bearing ruthenocene or ferrocene moieties and (**b**) bimetallic complex bearing a ferrocenyl–salicylaldimine moiety.

The organometallic complex containing ruthenocene (Compound 1, Figure 16a) was moderately active against MRSA, admittedly less so than the ferrocene derivative (Compound 2, Figure 16a). The organometallic complex containing ferrocene (2) (Figure 16a) was found to generate ROS, in contrast to 1, as indicated by oxidative stress assays. Consequently, the difference in activity was suggested to result from their differing abilities to generate ROS [142].

Incorporation of ferrocene as well as ruthenium in a half-sandwich heterobimetallic complex bearing a ferrocenyl–salicylaldimine moiety (Figure 16b) showed promising activity against *Mycobacterium tuberculosis*. Due to the observed glycerol-dependent antimycobacterial activity, a possible mechanism of action involves disruption of glycerol metabolism and accumulation of toxic intermediate metabolites. The complex was found to possess relatively low cytotoxicity in vitro against normal microbial flora, which also suggests selectivity [143].

A Ru(II)–Pt(II) bimetallic complex, [RuCl(tpy)(dpp)PtCl_2_](PF_6_), where dpp = 2,3- bis(2-pyridyl)pyrazine and tpy = 2,2’:6’,2’’-terpyridine, was reported to inhibit the growth of *E. coli* cells (albeit at a relatively high concentration of 400 µM). In contrast, its monometallic Ru(II) precursor, [RuCl(tpy)(dpp)](PF_6_), was inactive against *E. coli*. The improved activity of the Ru(II)/Pt(II) heteronuclear complex was attributed to the *cis*-PtCl_2_ moiety, although the heterobimetallic complex was still less effective than cisplatin alone [144]. Although [RuCl(tpy)(dpp)PtCl_2_](PF_6_) was reported in a follow-up study to induce DNA photocleavage, the effect of light irradiation on its antibacterial activity was not assessed [145].

### 5.4. Ruthenium-Based Carbon-Monoxide-Releasing Molecules (CORMs)

**Figure 17:**
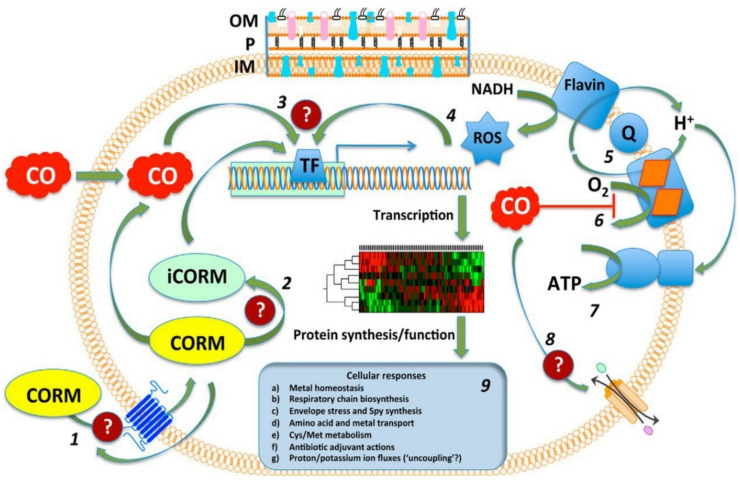
Modes of action and intracellular targets of CORMs. The bacterial membrane includes the inner membrane (IM), the outer membrane (OM), and periplasm (P), which are represented at the top. *1.* CORMs enter bacteria by unknown pathways and mechanisms; CO enters the cells by diffusion. *2.* After they enter the cell, CORMs release CO, forming inactivated CORM (iCORM). *3.* CO, CORM, and iCORM are detected by transcription factors (TFs), causing transcriptional changes. *4.* TFs are activated by ROS that may be generated directly by CORMs or can be generated as a result of the interaction of CORMs with the respiratory chain. *5.* A simplified aerobic respiratory chain of bacteria is represented, consisting of a flavin-containing NADH dehydrogenase, a ubiquinone (Q) pool, and a terminal heme-containing quinol oxidase. *6.* CO binds to the heme-containing quinol oxidase active site, competing with oxygen and impeding respiration. *7.* Impairment of ATP generation by ATP synthase. *8.* CO or CORM may directly or indirectly interact with IM transporters. *9.* Diverse cellular responses to CO and CORM. Question marks represent unknown targets, effects, or mechanisms: transport into (or out of) cells; intracellular mechanisms of CO release from CORMs; interaction with TFs and modification of gene expression by CORMs; effects of CORMs on membrane transporters. Figure reproduced from [146].

With a unique mode of action involving ligand exchange, carbon-monoxide-releasing molecules (CORMs) represent an emerging class of biologically active organometallic derivatives. Although their mechanism of action is fairly complex and not yet fully understood (Figure 17), CORMs release carbon monoxide (CO) to bind to intracellular targets, which is partially responsible for their activity. The chemistry and antimicrobial activity of ruthenium-based CORMs have already been reviewed in several excellent works [32,146,147,148,149]. The reader can find in the following pages a summary of what has already been reviewed, as well as references to more recent research.

**Figure 18:**
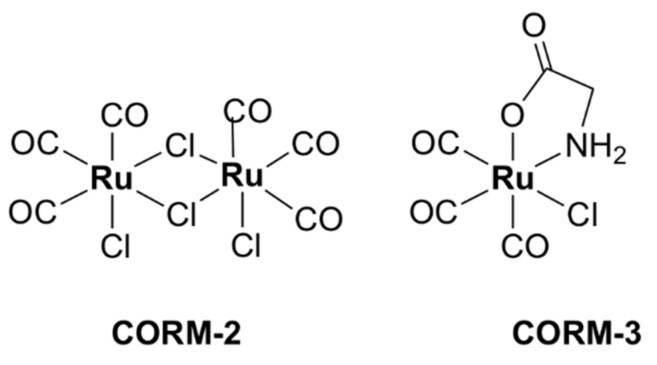
Chemical structures of CORM-2 and CORM-3.

CORM-2, a highly lipophilic dinuclear ruthenium(II) complex with the formula [Ru(CO)_3_Cl_2_]_2_ (Figure 18), and the water-soluble mononuclear CORM-3, [Ru(CO)_3_Cl(glycinate)] (Figure 18), have been intensely investigated over the last two decades. Various reports have shown that CORM-2 and CORM-3 exhibit broad-spectrum antibacterial activity against several strains and clinical isolates of Gram-positive (*S. aureus, Lactobacillus lactis*) and Gram-negative (*E. coli*, *H. pylori*, *Campylobacter jejuni*, *P. aeruginosa*, *Salmonella enterica* serovar Typhimurium) bacteria [63,64,66,67,150,151,152,153]. Notably, CORM-3 displays bactericidal activity against antibiotic-resistant *P. aeruginosa* [66], *H. pylori* [64], and *E. coli* [67] strains.

Studies in various buffers indicated that significant ligand exchange is likely to occur in biological media. Both the Cl‾ and glycinate ligands of CORM-3 are labile and undergo partial or full displacement by either water molecules or other counter-ions (e.g., phosphate) existing in the buffer or growth medium constituents [154].

#### 5.4.1. Mechanisms of Action

##### The Role of CO

CO is an inorganic compound that can bind hemoglobin with highly toxic effects. CO is produced endogenously as a result of heme breakdown by heme oxygenase. It is generally known to interact with metalloproteins due to its affinity towards transition metals (for instance, the ferrous ions in hemoglobin). Despite its notorious toxic effects, CO acts as a signaling molecule with important therapeutical properties, which include anti-inflammatory, anti-apoptotic and anti-proliferative effects [146]. The possibility of limiting its intrinsic severe toxicity and enhancing the biological activity has been explored with pro-drugs acting as CO-releasing molecules (CORMs), including transition metal (Mn, Ru, Fe, Mo) carbonyl complexes.

Ru-carbonyl CORMs were initially thought to act merely as vectors designed to deliver the toxic CO gas inside bacterial cells and, hence, the respiratory chain was presumed to be the main target of these molecules. The antibacterial activity of Ru-based CORMs was attributed to their ability to release CO in certain microenvironments of the cell, effecting an increase in the ratio of CO relative to O_2_, which eventually impedes the oxygen metabolism [35,142,150]. Indeed, there is substantial evidence in the literature that CORM-2 and CORM-3 impair aerobic respiration in *E. coli* [152,155,156], *P. aeruginosa* [66,157], *H. pylori* [64], *C. jejuni* [150], and *S. enterica* [152]. However, administration of BSA-Ru(II)(CO)_2_, an adduct formed between BSA and the hydrolytic decomposition products of CORM-3 in vitro, was demonstrated to release CO in a controlled manner in tumor-bearing mice, but did not produce any significant effect on bacterial growth in *E. coli* cells [158]. Additionally, in physiological conditions CORM-3 was found to release low amounts of CO inside bacterial cells (for 100 µM CORM-3, the concentration of CO detected in cells was < 0.1 µM) [159]. Thus, the toxicity of CO alone appears to be insufficient to explain the antibacterial activity of these compounds.

##### ROS Generation

ROS-induced oxidative stress has also been assessed as a possible mechanism of action responsible for the antimicrobial activity of CORMs. This assumption was based on the positive correlation observed in *E. coli* between the bactericidal activity and the ROS levels generated upon treatment with CORMs [35,160]. In vitro studies performed in aqueous solutions indicated that CORM-2 and CORM-3 are able to generate OH• [160,161] and O_2_^•^‾ [151,161] radicals. However, the amount of superoxide ions was measured to be only ~1% of the total CORM-3 concentration, which does not account for the bactericidal activity of the compound [151]. In airway smooth muscle cells, CORM-2 stimulated ROS production through inhibition of cytochromes on both NAD(P)H oxidase and the respiratory chain [162,163]. Furthermore, *E. coli* mutant strains in which genes encoding catalases and superoxide dismutases (SODs) have been deleted are more susceptible to CORM-2 treatment due to an increase in intracellular ROS content; this effect is alleviated upon supplementation of the culture medium with antioxidants (reduced glutathione or cysteine) [160]. For CORM-3, however, addition of catalase or SOD did not have any significant impact on its respiratory effects in *E. coli*, implying that peroxide or superoxide are not involved in the activity of CORM-3 in these cells [152,155]. In *C. jejuni*, however, CORM-3 was shown to inhibit respiration and generate hydrogen peroxide, although no effect on cell growth was observed even at concentrations as high as 500 µM [150]. Addition of various sulfur-containing antioxidants, namely cysteine, N-acetyl cysteine (NAC), or glutathione (GSH), abolished the respiratory and growth inhibitory effects of ruthenium–carbonyl CORMs in *E. coli* and *P. aeruginosa* [66,151,157,160,164]. However, this effect is presumed to be independent of the antioxidant activity of CORMs, based on two reports showing that NAC strongly inhibits the uptake of CORMs in *E. coli* cells [155] and a NAC–CORM-2 complex displays no activity against bacterial cells [165]. It is more likely that the Ru(II) species derived from CORMs in biological environments form adducts with exogenous compounds bearing thiol groups, which cannot be readily internalized into bacteria and are therefore less potent antibacterial agents. Non-thiol antioxidants do not alleviate the inhibitory effects of CORMs on respiration [155]. Moreover, CORM-3 was shown to impair the tricarboxylic acid (TCA) cycle, also known as the Krebs cycle, in *E. coli* cells treated under anaerobic conditions, suggesting that its activity extends beyond ROS generation [67]. Hence, it is unlikely that ROS-induced oxidative stress represents the main mechanism behind the CORMs’ bactericidal activity, although ROS generation probably plays some part in inhibiting the growth and respiration of CORM-2 on *E. coli* cells.

##### Membrane Damage

The bactericidal activity of CORM-3 has also been linked to membrane damage in *E. coli* cells, as penetration of propidium iodide [156] and N-phenyl-1-napthylamine [166], fluorescent dyes that cannot pierce healthy membranes, is allowed after CORM-3 treatments. Clearly, loss of membrane integrity can occur in the aftermath of cell death; therefore, it is not necessarily part of the antibacterial mechanism.

##### The Role of the Ru(II) ion Interactions with Proteins and DNA

In ruthenium-based CORMs, the Ru ion was assumed to have more of a structural role. This paradigm was based on the assumption that ruthenium–carbonyl CORMs were stable enough to reach the intracellular environment, where reducing agents (e.g., sulfites) would trigger CO release [35]. However, more recent research suggests that CORM-2 and CORM-3 undergo ligand exchange and interact with serum proteins in vivo to form protein–Ru(CO)_2_ adducts. CO release occurs following decomposition of these adducts [158,167,168,169,170]. Additionally, no CO release was detected in vitro upon addition of CORM-2 and CORM-3 in phosphate buffers and cell culture media in the absence of sulfur-containing reducing agents [159].

Therefore, CO release cannot be solely responsible for the cytotoxic effects of CORMs, which is further inferred by the fact that CORM-3 is toxic even for heme-deficient cells [166]. Moreover, Ru-carbonyl CORMs are considerably more active than other non-ruthenium-based CORMs [63,102,157] and inhibit aerobic respiration and bacterial growth more potently than CO gas alone [66,156]. Taking into account all of the above-mentioned arguments, it stands to reason that the ruthenium ion plays an essential role in the antimicrobial activity of these metal complexes.

The Ru(II) ion in CORM-3 was found to bind tightly to thiols. Addition of various compounds containing thiol groups in growth media protected both bacterial and mammalian cells against CORM-3. The binding affinities of CORM-3 for the compounds tested vary in the order cysteine ≈ GSH >> histidine > methionine. Moreover, a direct positive correlation was found between the protective effects of these compounds and the dissociation constants of the complexes formed between CORM-3 and the respective thiol compounds. Other amino acids (alanine and aspartate) did not exert significant protective effects. Southam et al. suggest a mechanism in which CORM-3 undergoes ligand displacement reactions in buffers or media to generate complex species in which the Ru(II) centers are readily available to bind to intracellular components such as glutathione. Another mode of action for CORMs is therefore presumed to involve Ru(II) binding to intracellular targets, impairment of glutathione-dependent systems, and disruption of redox homeostasis [159].

Indeed, CORMs have been shown to interact with various intracellular or membrane-bound proteins. CORM-3 has been shown to interact in vitro with the serum proteins myoglobin, hemoglobin, transferrin, and albumin, forming protein–Ru(II)(CO)_2_ adducts [167,168]. As described above, CORM-3 possesses two labile ancillary ligands (Cl‾ and glycinate), which can be readily released in aqueous media, allowing further interaction with serum proteins to occur [168]. With BSA, CORM-3 forms in vitro a [BSA-(Ru(II)(CO)_2_)_16_] complex, in which the Ru(II)(CO)_2_ adducts bind to histidine residues exposed on the surface of the protein. As stated above, the CO-releasing protein–Ru(II)(CO)_2_ complex did not have any significant effect on bacterial growth in *E. coli* cells [158]. The reason is unknown. In addition, CORM-2 has also been shown to inhibit urease activity in *H. pylori* [64] and lactate dehydrogenase in primary rat cardiocytes [171]. *H. pylori* urease is essential to the survival of the bacterium in the acidic gastric milieu [172]; therefore, its inhibition can represent a viable strategy against *H. pylori* infections. The histidine-rich active site involved in coordination of Ni(II) ions is presumed to be the target of CORM-2. It is uncertain whether urease inhibition occurs via direct binding of the Ru(II) ion to the active site accompanied by Ni(II) displacement, or CO binding to the Ni(II) ion in the active site.

Soft and borderline transition metals have been shown to bind to Fe–S clusters, which are important cofactors of various enzymes including several pertaining to the Krebs (or TCA) cycle [173]. CO is also reported to bind to iron–sulfur clusters in a redox-dependent manner [174]. Therefore, Fe–S enzymes have been studied as potential targets for CORMs. Indeed, treatment of *E. coli* cells with CORM-2 resulted in an increase in intracellular iron, suggesting degradation of the Fe–S clusters. This assumption was further supported by the significant inhibition of two Fe–S proteins, aconitase B and glutamate synthase, following exposure of *E. coli* extracts to CORM-2. Although the presence of intracellular Fe–S clusters was shown to correlate with the antimicrobial activity of CORM-2, it was not clearly determined whether the Ru(II) ion of CORM-2 binds directly to Fe–S clusters, or if the degradation of the clusters occurs indirectly as a result of other processes [160]. However, a cell extract from *E. coli* overexpressing aconitase B displayed a 50% decrease in the activity of the enzyme after incubation with CORM-3, relative to untreated cells, suggesting that the protein–CORM-3 complex occurs at a post-translational level. Additionally, recent metabolomics studies in *E. coli* cells revealed that CORM-3 inhibits the activity of several Fe–S proteins, namely the glutamate synthase GOGAT and enzymes of the TCA cycle (aconitase B, isocitrate dehydrogenase, and fumarase). In response to the severe imbalance in the energy and redox homeostasis caused by the Ru-carbonyl complex, activation of the glycolysis pathway was detected in the CORM-3-stressed cells. Notably, other non-CO-releasing Ru(II) species, used as controls, were non-toxic to *E. coli* cells and had no effect on the Fe–S enzymes at the concentration used in this study (120 µM—a growth inhibitory but nonlethal concentration of CORM-3) [67].

Although numerous cytotoxic ruthenium complexes developed as anticancer agents have been shown to interact with DNA, no in vitro or in vivo studies clearly demonstrate whether Ru-based CORMs bind directly to DNA or not. However, CORM-2 has been shown to induce DNA damage and increase the expression of a double-strand break repair gene, *recA*, in *E. coli* [65,160]. DNA damage can be the result of CORM-2-induced generation of intracellular ROS, although this has not been clearly established [65].

##### Effects on Gene Expression

Transcriptome studies on *E. coli* revealed that CORM treatments under either aerobic or anaerobic conditions trigger complex transcriptional responses of gene expression [151,156,166,175,176] that exceed those induced by CO alone [177]. CORM-2 and CORM-3 downregulate genes involved in aerobic respiration, energy metabolism, and biosynthesis pathways and upregulate those involved in the SOS response and DNA damage and repair mechanisms. A recent gene profiling study analyzed the effects induced by CORM-2 exposure on a multidrug-resistant extended-spectrum beta-lactamase (ESBL)-producing uropathogenic *E. coli* clinical isolate [65]. Numerous genes encoding the NADH dehydrogenase complex were repressed by CORM-2 [65], as was previously shown for CORM-3 in the *E. coli* K12 strain [156]. Transcriptomics analysis of *E. coli* cells treated with CORM-3 indicated altered expression of the cytochrome genes *cyoABCDE* and *cydAB* [151,156]. However, CORM-2 had no effect on the expression of cytochrome genes, which could be attributed to the differences in the growth media [65].

Exposure to CORM-2 and CORM-3 increased the expression of genes coding for proteins with roles in stress response and adaptation, e.g., *ibBA*, *ibpA,* and *spy* [65,151,156,166,176]. The *spy* gene appears to be one of the main non-heme targets for CORMs. Several genes coding for multidrug efflux pump proteins were also upregulated by CORM-2 [65] and CORM-3 [166]. Upregulation of multidrug efflux pump systems has been shown to lead to the development of resistant phenotypes over time [178]. However, the growth inhibitory activity of CORM-2 was not diminished by repeated exposure (20 times), neither in the multidrug-resistant ESBL-producing *E. coli* strain, nor in two other antibiotic-susceptible *E. coli* strains [65].

Significant upregulation has also been found for genes involved in metal homeostasis, such as iron or zinc [151,156,166], and genes involved in the uptake and/or metabolism of sulfur compounds (sulphate-thiosulphate, methionine, cysteine, glutathione) and the sulfur starvation response [67,151,166,176]. In agreement with the already-discussed inhibitory effects of CORMs on Fe–S enzymes [67,160], genes involved in Fe–S cluster biosynthesis and repair are also upregulated by CORM-2 and CORM-3 [151,166,176]. Transcriptomic data, therefore, correlate well with the in vitro observation that sulfur species represent intracellular targets of Ru(II)-based CORM [151].

#### 5.4.2. Ruthenium-Based CORM Polymers

**Figure 19:**
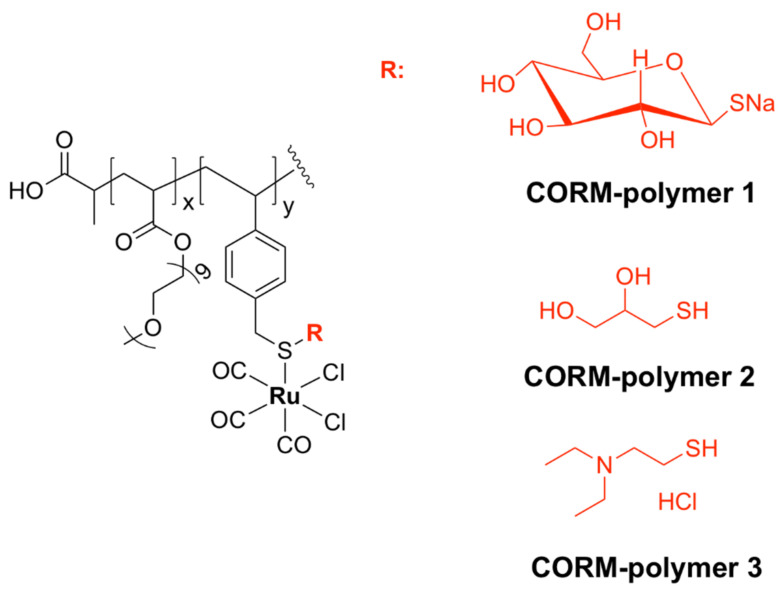
Chemical structures of ruthenium-based CORM-polymers 1–3.

Encapsulation of drugs into polymers is a modern therapeutic strategy that makes use of building blocks with 3D structures that enable controlled ligand exchange [179]. Conjugation to lipophilic polymers reduces the access of water molecules to CORMs, causing the solvent-assisted ligand exchange reactions to occur at a slower, sustainable pace. A Ru-based CORM was conjugated to the side chain of polymeric fibers bearing different thiol moieties, yielding the three water-soluble CO-releasing macromolecules CORM-polymers 1–3 (Figure 19). The resulting polymers have been shown to exhibit bactericidal activity against *P. aeruginosa* and to prevent biofilm formation more efficiently than CORM-2, most likely due to their high CO-loading capacity, controlled release of CO, and prolonged half-lives. Notably, the antimicrobial activity was not directly proportional to the half-lives of the complexes, since CORM-polymer 2 was the most active compound of the series, while CORM-polymer 1 had the longest half-life [180].

#### 5.4.3. Cellular Uptake

The mechanisms of uptake of CORM-2 and CORM-3 complexes are unknown. It is also unclear which are the Ru-CORM-derived species that pierce the bacterial membranes and it is likely that different species use different mechanisms of uptake. However, ruthenium species (quantified using ICP-MS) have been found to accumulate to high levels in *E. coli* cells treated with either CORM-2 or CORM-3 [155,156]. CORM-3 was found to be rapidly taken up by *E. coli* cells at an initial rate of 85 µM/min over the first 2.5 minutes after treatment, with intracellular Ru levels reaching a plateau after ∼40 min [151]. CORM-3 accumulated to higher levels in *S. enterica* serovar Typhimurium than in *E. coli*, and at a faster initial uptake rate (>120 µM/min). This may explain, at least partially, why *Salmonella* strains are more susceptible to CORM-3 than *E. coli* [153]. Notably, simultaneous addition of NAC and CORM-2 or CORM-3 reduced the ruthenium accumulation inside bacterial cells, which is probably why exogenous thiols, such as NAC, are able to interfere with the antibacterial activity of both CORM-2 and CORM-3 [155]. These findings also suggest that the bactericidal effects of these compounds are dependent on the ruthenium uptake by the bacterial cells [151]. It has been suggested that Ru–based CORMs could be transported actively, or diffuse, inside the cells via an unidentified route against the concentration gradient and, due to the reactions that occur inside the cells, uptake can continue passively [153].

#### 5.4.4. Toxicity and Pharmacokinetics

The more lipophilic, DMSO-soluble, CORM-2 is generally more toxic to mammalian cells than the water-soluble CORM-3. It has been suggested that the use of DMSO is at least partially accountable for the increased cytotoxicity [102,171,181]. Toxic concentrations reported for eukaryotic cells are considerably higher than the MIC values [66,102,153,164,169,170,171,181]. For instance, the bactericidal effects against *P. aeruginosa* occurred at concentrations of CORM-3 that are 50-fold lower than the toxic concentrations for macrophages [66]. However, survival of the mammalian cells was drastically increased by the presence of exogenous thiols in the growth media. For instance, treatment with 25 µM CORM-3 in phosphate-buffered saline (PBS) decreased survival relative to untreated human colon carcinoma RKO cells in PBS by 92%, compared with only 23% in RPMI-1640 growth medium, whilst in DMEM the survival rate was enhanced relative to untreated controls [159].

In vivo studies revealed that a two-week CORM-3 treatment (with increasing doses from 7.5 to 22.5 mg/kg) caused no mortality or any apparent side effects to healthy mice [66]. In contrast, consecutive administrations of 15–37 mg CORM-3/kg in rats caused severe liver and kidney damage after 21 days of treatment. Biodistribution studies in CORM-3-treated mice concluded that the ruthenium species derived from CORM-3 mostly accumulated in the blood for the first hour after the intravenous administration and then were slowly distributed to the kidneys, liver, lungs, and heart. Notably, only trace amounts of ruthenium were found in the brain, suggesting that the complex and its derived species did not cross the blood–brain barrier. Both ruthenium and elevated levels of protein were found in the urine of the CORM-3-treated mice, indicating kidney damage. Moreover, the Ru^II^ center was oxidized to Ru^III^ in vivo by enzymes such as cytochrome P450 [170].

#### 5.4.5. In Vivo Studies Regarding the Antibacterial Activity of CORMs

Several studies have reported the in vivo antibacterial activity of Ru–carbonyl CORMs [66,157,182]. In a murine model of polymicrobial sepsis, treatment with 10 μM CORM-2/kg 12 and 2 h before the inoculation of bacteria resulted in a significant decrease in bacterial counts relative to the vehicle-treated mice. CORM-2 improved the survival rates of heme oxygenase (HO)-1 null mice, mutants that are more susceptible to polymicrobial infection, even when administered intraperitoneally after the onset of sepsis [182]. Administration of CORM-2 (12.8 mg/kg) was also shown to significantly increase the survival rates of BALB/c mice infected with *P. aeruginosa* [157].

Injections of CORM-3 (7.5–22.5 mg/kg) in the murine model of *P. aeruginosa* infection reduced bacterial counts in the spleen and increased the survival rates of the infected mice (from 20% in the vehicle-treated group to 100% in the CORM-3-treated mice). Moreover, treatment with CORM-3 reduced bacterial counts in the spleen of immunosuppressed mice to a similar degree to in immunocompetent mice, suggesting a direct, rather than host-mediated, antibacterial effect of CORM-3 [66].

The modes of action in vivo of these compounds are still unknown and require further assessment. The promising results of these studies, however, pave the way for a more extensive preclinical evaluation of the antibacterial efficacy of Ru-based CORMs.

### 5.5. Ruthenium Complexes in Antimicrobial Photodynamic Therapy

Photodynamic therapy (PDT) is a therapeutic strategy that makes use of a combination of photosensitive molecules, light, and molecular oxygen. PDT has been investigated against a range of medical conditions, including atherosclerosis, psoriasis, and malignant cancers [183,184]. Antimicrobial photodynamic therapy (aPDT) has been used against a variety of microbial pathogens (bacteria, fungi, and viruses). It relies on the ability of a compound, a photosensitizer, to generate singlet oxygen (^1^O_2_) and other ROS upon light irradiation, causing bacteria inactivation [185].

Ru(II) complexes, particularly Ru(II)–polypyridyl complexes, have been intensively investigated for PDT applications against malignant cancers due to their optical properties, such as the long luminescence lifetimes of the triplet metal-to-ligand charge transfer (MLCT) excited state [184,186]. The remarkable potential of Ru complexes as PDT agents has been confirmed by TLD-1433 [18], which is currently undergoing phase II clinical studies as a photosensitizer for PDT against bladder cancer.

**Figure 20:**
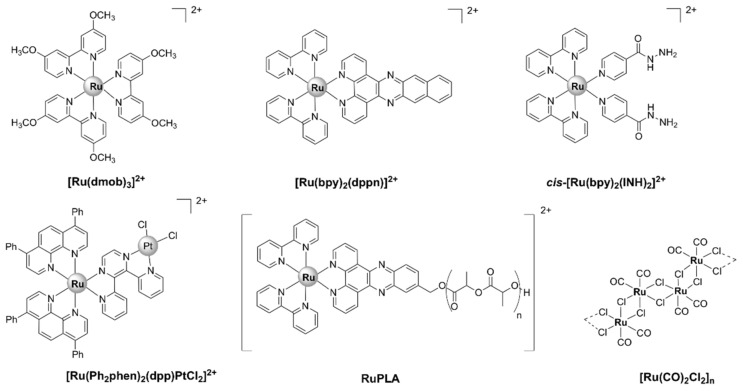
Chemical structures of ruthenium complexes developed for Antimicrobial Photodynamic Therapy.

Taking into account the remarkable success of Ru(II)-based PDT agents in the treatment of cancer, several Ru(II) complexes have been considered as potential photosensitizers for aPDT. For instance, [Ru(dmob)_3_]^2+^ (Figure 20), where dmob = 4,4’-dimethoxy-2,2’-bipyridine, was more active than the corresponding complexes bearing bpy and phen ligands against *S. aureus*, *P. aeruginosa,* and *C. albicans* strains. The enhanced activity was attributed to the increased lipophilicity of the complex due to the presence of methoxy groups in its structure, which can translate to enhanced uptake by the bacterial cells [68].

[Ru(bpy)_2_(dppn)]^2+^ (Figure 20), where bpy = 2,2’-bipyridine and dppn = 4,5,9,16-tetraazadibenzo[*a,c*]-napthacene, was shown to cause potent photoinactivation of *E. coli* cells, while dark incubation with the compound had no effect on the viability of the microorganism. Treatment with [Ru(bpy)_2_(dppn)]^2+^ led to a 70% CFU decrease at 0.1 µM and complete inactivation at 0.5 µM following light activation [187].

The ruthenium(II) complex *cis*-[Ru(bpy)_2_(INH)_2_]^2+^ (Figure 20), where INH = isoniazid, has been shown to undergo stepwise photoactivation in aqueous media after irradiation with 465 nm blue light. The resulting products of this process are two equivalents of the antituberculosis drug isoniazid and *cis*-[Ru(bpy)_2_(H_2_O)_2_]^2+^. *cis*-[Ru(bpy)_2_(INH)_2_]^2+^ was inactive in the dark; however, upon photoactivation, it was 5.5-fold more efficient against *Mycobacterium smegmatis* in comparison with isoniazid. Notably, *cis*-[Ru(bpy)_2_(INH)_2_]^2+^ displayed high selectivity towards mycobacteria over healthy MRC-5 human lung cells in vitro [69].

A heterobimetallic complex [Ru(Ph_2_phen)_2_(dpp)PtCl_2_]^2+^ (Figure 20), where Ph_2_phen = 4,7-diphenyl-1,10-phenanthroline and dpp = 2,3-bis(2-pyridyl)pyrazine, has been reported to induce photocytotoxic effects in *E. coli* cells in the presence of oxygen and visible light. The dose required for complete cell growth inhibition under visible light irradiation was 5 µM, as opposed to 20 µM in the dark [70]. In comparison, cisplatin induced complete cell growth inhibition at 5 µM in the dark, but a similar complex, [RuCl(tpy)(dpp)PtCl_2_]^+^ (see above), had the same effect at 200 µM [145]. Inside the cells, photoactivated [Ru(Ph_2_phen)_2_(dpp)PtCl_2_]^2+^ was shown to bind to chromosomal DNA [70]. Further experiments are needed to assess the nature of the DNA binding, as well as what species are responsible for the activity.

Incorporation in or conjugation with biocompatible polymers has been used as an efficient strategy to increase the ability of ruthenium complexes to penetrate bacterial cell walls and therefore their antimicrobial activity. A Ru(II)–polypyridyl complex, [Ru(bpy)_2_-dppz-7-hydroxymethyl][PF_6_]_2_ (RuOH), where bpy = 2,2’-bipyridine and dppz = dipyrido[3,2-*a*:2;2’,3’-*c*]phenazine, was found to be inactive against Gram-positive and Gram-negative bacteria. This lack of activity was thought to stem from its low uptake by bacterial cells. In order to solve this issue, RuOH was conjugated to the end-chain of a hydrophobic polylactide (PLA) polymer to form ruthenium–polylactide (RuPLA) nanoconjugates (Figure 20). Although RuPLA nanoconjugates displayed superior photophysical properties, including luminescence and enhanced ^1^O_2_ generation, they were only moderately active against Gram-positive (*S. aureus*, *S. epidermidis*) bacteria, with MIC values of 25 µM. The RuPLA nanoconjugates remained non-toxic to the Gram-negative (*E. coli* and *P. aeruginosa*) bacterial strains and displayed phototoxicity against human cervical carcinoma cells (IC_50_ = 4.4 µM) [185].

In a recent study, the antibacterial activity of the purely inorganic polymer [Ru(CO)_2_Cl_2_]_n_ (Figure 20), with repeating dicarbonyldichlororuthenium (II) monomers, was studied against *E. coli* and *S. aureus*. Significant inhibitory effects were observed on both strains at concentrations as low as 6.6 ng/mL after irradiation with 365 nm UV light. Interestingly, the polymer displayed stronger photobactericidal activity against the Gram-negative *E. coli* (MIC ~33 ng/mL) than the Gram-positive *S. aureus* (MIC ~166 ng/mL) bacteria. In addition, [Ru(CO)_2_Cl_2_]_n_ remained non-toxic to human dermal fibroblasts and red blood cells at concentrations much higher than the MIC values. Although the complex was considerably toxic to both bacterial strains in the dark, the antibacterial activity of [Ru(CO)_2_Cl_2_]_n_ was significantly increased upon photoirradiation, which can be attributed to the enhanced generation of ROS under UV light. SEM analysis revealed that its mode of action might involve disruption of bacterial membranes. Moreover, [Ru(CO)_2_Cl_2_]_n_ was able to cause morphological changes to biofilm structures and to disassemble the biofilm matrix [71]. It should be noted that although the structure of [Ru(CO)_2_Cl_2_]_n_ is similar to that of CORMs, there is no information available in the literature on the ability of the polymer to release CO or undergo ligand exchange in aqueous media.

**Figure 21:**
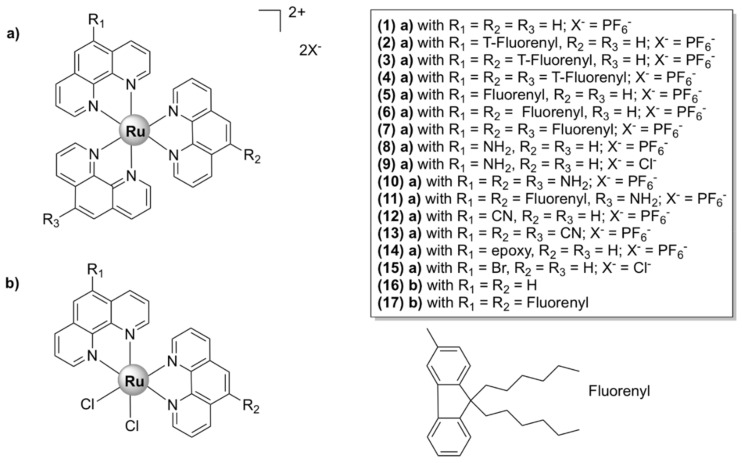
Chemical structures of the homo- or heteroleptic polypyridyl Ru(II) complexes **(1)**–**(17)** with the general formulae [Ru(Phen)_3_](PF_6_)_2_, [Ru(Phen)_2_(Phen-X)](PF_6_)_2_, [Ru(Phen)(Phen-X)_2_](PF_6_)_2_, [Ru(Phen-X)_3_](PF_6_)_2_, [Ru(Phen-X)_2_Cl_2_], or [Ru(Phen)_2_Cl_2_]. The core structures of the complexes **(1)**–**(17)** correspond to either (**a**) or (**b**), as denoted in the top right corner of the figure. The fluorene unit was bonded to the 1,10-phenanthroline moiety ligand either directly (Fluorenyl, bottom right corner) or via a triple bond (T-Fluorenyl).

The antibacterial photosensitizing activity towards a panel of bacterial strains has been assessed for seventeen homo- or heteroleptic polypyridyl Ru(II) complexes with the following formulae: [Ru(Phen)_3_](PF_6_)_2_, [Ru(Phen)_2_(Phen-X)](PF_6_)_2_, [Ru(Phen)(Phen-X)_2_](PF_6_)_2_, [Ru(Phen-X)_3_](PF_6_)_2_, [Ru(Phen-X)_2_Cl_2_], or [Ru(Phen)_2_Cl_2_] (Figure 21), varying due to the number and the nature of the substituents. With regard to the most active complexes, **2**, **5**, and **6** stood out, **5** was highly efficient against MRSA N315 even without light irradiation, and **2** demonstrated activity against four *S. aureus* strains, one *E. coli* strain, and three *P. aeruginosa* strains. However, **2** and **5** were more toxic towards eukaryotic cells upon light irradiation, with **6** being non-toxic. The counterion (PF_6_‾ vs. Cl‾) did not appear to have a significant effect on the antibacterial activity. In contrast, a dicationic charge was vital to the activity, taking into account that the two neutral Ru(II) complexes, **16** and **17**, were inactive. Surprisingly, the best photosensitizers for ^1^O_2_ production (**8**, **9**, **10**, **15**) did not correspond to the most efficient aPDT agents (**2**, **5**, **6**). The ability of the complexes to interact efficiently with bacteria seems to be crucial for aPDT activity, considering the short half-life of ROS generated upon light irradiation. Thus, solely increasing ^1^O_2_ production is not sufficient to yield more efficient aPDT agents. Parameters impacting the interactions with bacteria, such as lipophilicity and ability to form aggregates, should also be considered in the development of optimized future compounds for aPDT [184].

## 6. Antiparasitic Activity of Ruthenium Complexes

Parasitic infections, including malaria (*Plasmodium* sp.), Chagas’ disease (*Trypanosoma cruzi*), African trypanosomiasis (*Trypanosoma brucei*), and leishmaniasis (*Leishmania* sp.), mainly affect the tropical and subtropical regions of Africa and Asia and only a narrow spectrum of effective drugs is available for treatment. In this context, several ruthenium complexes have been reported as efficient antiparasitic agents active against malaria, leishmaniasis, trypanosomiasis, and Chagas’ disease [188]. Generally, the enhanced antiplasmodial activity of these complexes when compared to the free ligands is thought to be related to their increased lipophilicity, which translates to increased uptake into the parasite’s cells and/or increased ability to evade the parasite’s drug efflux pumps.

### 6.1. Antiplasmodial Activity

Malaria is a highly infectious parasitic disease, with over 40% of the world’s population living in an endemic region. Malaria parasites belong to the genus *Plasmodium*, the most virulent strain being *P. falciparum* [189,190]. Conventional treatment strategies use either quinoline-based drugs, such as chloroquine (CQ)) and its derivatives, or fixed-dose combination therapies containing a derivative of the Chinese natural product artemisinin. The increasing widespread resistance to these compounds requires urgent attention to the development of new therapeutic strategies [190,191].

**Figure 22:**
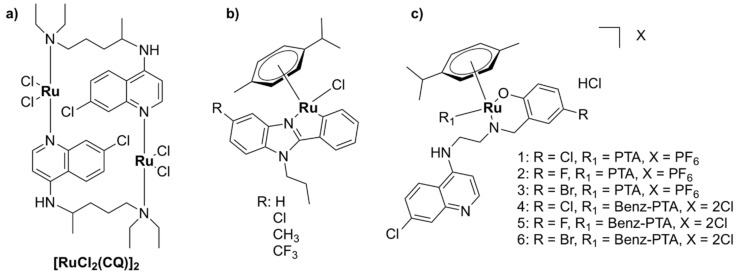
Chemical structures of ruthenium complexes with antiplasmodial activity. (**a**) [RuCl_2_(CQ)]_2_, (**b**) cyclometallated Ru(II) complexes of 2-phenylbenzimidazoles, and (**c**) PTA-derived ruthenium(II) quinoline complexes.

An organometallic complex, [RuCl_2_(CQ)]_2_ (Figure 22a), where CQ = chloroquine, displayed 2–5-fold increased activity against *P. falciparum* compared with chloroquine diphosphate in vitro [192]. Moreover, the complex was significantly more active when compared with its organic derivative in mice infected with *Plasmodium berghei*, with no apparent signs of acute toxicity up to 30 days after treatment [193]. [RuCl_2_(CQ)]_2_ was shown to bind to hematin and inhibit aggregation of β-hematin (synthetic hemozoin—a target of the malaria parasite) in vitro, albeit to a slightly lower extent than chloroquine diphosphate. However, the heme aggregation inhibitory activity of the complex is significantly higher than that displayed by chloroquine, suggesting that the main target of the complex is the heme aggregation process. [RuCl_2_(CQ)]_2_ was shown to be significantly more lipophilic than chloroquine diphosphate, suggesting that the addition of Ru(II) induced drastic changes in the pharmacokinetic profile of the organometallic compound. One chlorido ligand from each of the two Ru(II) centers is displaced by water molecules upon addition to aqueous solutions. The resulting species, [RuCl(OH_2_)_3_(CQ)]_2_[Cl]_2_, is considered to be the active species in vitro and in vivo. The enhanced activity of the complex against CQ-resistant strains of *P. falciparum* was suggested to relate to its lipophilicity. This can be explained by the fact that the parasite efflux pump, usually involved in the resistance mechanism to chloroquine, has a lower ability to bind to highly lipophilic drugs [192].

CQ has also been used as a chelating ligand in a series of organoruthenium complexes with the general formula [RuCQ(η^6^-C_10_H_14_)(N–H)]^2+^, where η^6^-C_10_H_14_ is *α*-phellandrene and N–H is either 2’-bipyridine (BCQ), 5,5’-dimethyl-2,2’-bipyridine (MCQ), 1,10-phenanthroline (FCQ), or 4,7-diphenyl-1,10-phenanthroline (FFCQ). As was previously shown for [RuCl_2_(CQ)]_2_, the Ru–CQ bonds are stable, and CQ is not released upon aquation. The organoruthenium complexes displayed intraerythrocytic activity against CQ-sensitive and -resistant strains of *P. falciparum*. Unlike CQ, the complexes exerted moderate activity against the liver stage and potent activity against the sexual stage of the parasite, suggesting that they operate via a different mechanism than that of CQ. It has been shown that [RuCQ(η^6^-C_10_H_14_)(N–H)]^2+^ induces oxidative stress in the parasite, which might be linked to their mode of action. In addition, the organoruthenium complexes displayed low mammalian cytotoxicity and inhibited parasitemia in mice infected with *P. berghei* [194].

A range of Ru(II)–arene complexes were developed in the knowledge that increasing the lipophilic properties of a drug is likely to increase passive diffusion through membranes and hence the antiplasmodial activity. For instance, a series of half-sandwich Ru(II) complexes with aryl-functionalized organosilane thiosemicarbazone ligands were more active against *P. falciparum* at low micromolar concentrations (2.29–6.66 µM) and less cytotoxic to the Chinese Hamster Ovarian (CHO) cell line in comparison with the corresponding free ligands. It should be stated that the activity of the complexes was still much lower than that of both CQ and artesunate, which were used as controls. However, the complexes also displayed much lower resistance index values relative to the control drugs, which suggests that the parasites are less likely to develop cross-resistance to the metal complexes [195].

An enhancement of the antiplasmodial activity has also been observed for cyclometallated Ru(II) complexes of 2-phenylbenzimidazoles (Figure 22b), when compared with the free ligands. These complexes were found to be active against CQ-sensitive and multidrug-resistant *P. falciparum* strains, with IC_50_ values in the low to submicromolar range (0.12–3.02 µM). The nature of the substituent on the η^6^-*p*-cymene moiety does not seem to influence the activity to a great extent. Although CQ was still more active than the cyclometallated complexes against both strains, the latter displayed lower resistance index values relative to CQ. In addition, the metal complexes displayed relatively low cytotoxicity against the mammalian CHO cells. Notably, the Ru(II) complexes were found to be more active than the Ir(III) analogues on the resistant strain [196], which was also reported for other Ru(II)–arene complexes [197]. PTA-derived ruthenium(II) quinoline complexes (Figure 22c) were, however, generally less effective against CQ-sensitive and resistant strains of *P. falciparum* than their Ir(III) correspondents, but were also much less toxic to the CHO cells. In addition, these RAPTA-like complexes inhibited β-hematin formation, suggesting that their mechanism of action is similar to that of CQ [198].

**Figure 23:**
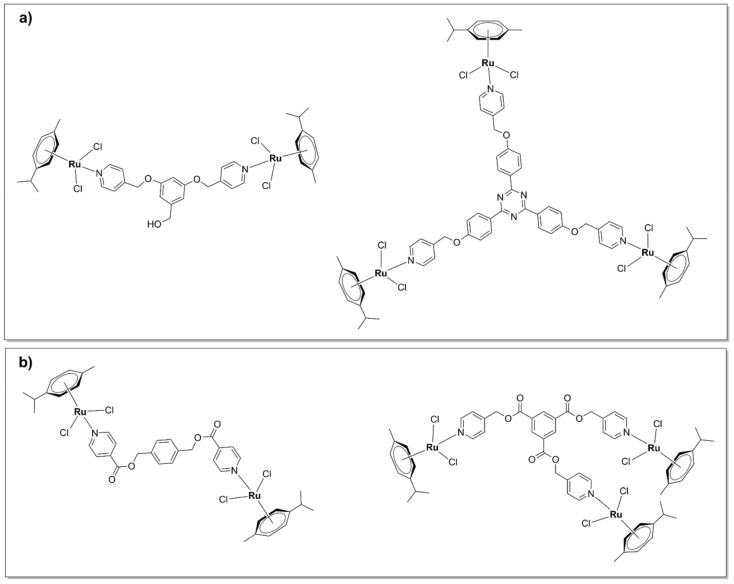
Di- and tri- nuclear Ru(II)-η^6^-*p*-cymene complexes in which the ruthenium centers are bridged by (**a**) pyridyl aromatic ether ligands and (**b**) pyridyl aromatic ester ligands.

Di- and tri- nuclear Ru(II)-η^6^-*p*-cymene complexes (Figure 23a), in which the ruthenium centers are bridged by pyridyl aromatic ether ligands, were evaluated against CQ-sensitive and -resistant *P. falciparum* strains. While the dinuclear derivative displayed only moderate activity, the trinuclear complex proved to be highly active in both strains, displaying activities in the nanomolar range (IC_50_ = 240 nM and 670 nM for the CQ-sensitive and -resistant *P. falciparum* strains, respectively). The trinuclear complex was also able to inhibit more efficiently β-hematin formation in vitro, in comparison with the dinuclear derivative, which suggests that hemozoin might be a target of the complexes in vivo. Notably, the trinuclear Ru(II) complex was only slightly more toxic than the corresponding tripyridyl ether ligand, indicating that it was the incorporation of a triazine moiety that had a more significant impact on activity [197]. This was confirmed by the fact that trinuclear Ru(II)-η^6^-*p*-cymene complexes, in which the ruthenium centers are bridged by pyridyl aromatic ester ligands lacking the triazine moiety (Figure 23b), are much less efficient antiparasitic agents [199].

**Figure 24:**
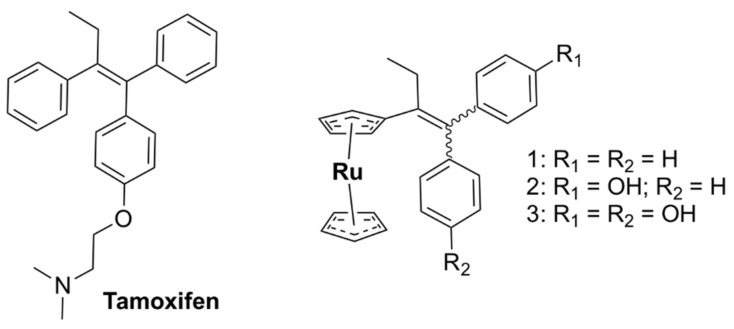
Chemical structures of tamoxifen and the ruthenocenyl complexes incorporating tamoxifen-based ligands.

Using ‘old’ drugs to assist in the search for new agents that are more efficient for either common or rare diseases is the scope of a relatively new therapeutic strategy called drug repositioning/repurposing. For instance, a series of ferrocenyl and ruthenocenyl derivatives incorporating tamoxifen-based compounds were tested against CQ-resistant *P. falciparum* blood forms. Tamoxifen (Figure 24) is an anticancer agent used in current treatment plans to prevent and treat breast cancer. The results within this series indicated that the ruthenocenyl-containing complexes (Figure 24) were more active than their ferrocenyl analogues, but still only displayed moderate activity (IC_50_ = 4.7–16.5 mM) against *P. falciparum*. The ruthenocenyl complexes were considered nontoxic to HepG2 cells [200].

### 6.2. Antitrypanosomal Activity

Chagas’ disease (American trypanosomiasis) affects millions of people worldwide, mainly in Central and South America, where the disease is endemic. It is a life-threatening disease caused by the parasite *Trypanosoma cruzi*. No vaccines are currently available, and treatment options are limited to only two drugs, benznidazole and nirfurtimox [201]. Sleeping sickness (African trypanosomiasis) predominantly affects people living in sub-Saharan Africa and is transmitted by the bite of the tsetse fly. The disease is caused by the insect-borne *T. brucei* parasite [202].

**Figure 25:**
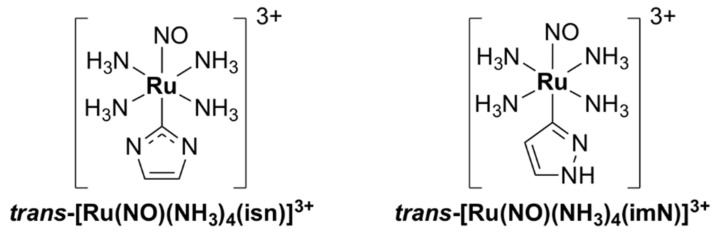
Chemical structures of the ruthenium NO-donor complexes *trans*-[Ru(NO)(NH_3_)_4_(isn)]^3+^ and *trans*-[Ru(NO)(NH_3_)_4_(imN)]^3+^.

Two Ru(II)–NO donor compounds, namely *trans*-[Ru(NO)(NH_3_)_4_(isn)](BF_4_)_3_ (Figure 25) where isn = isonicotinamide and *trans*-[Ru(NO)(NH_3_)_4_(imN)](BF_4_)_3_ (Figure 25) where imN = imidazole, displayed significant activity against *T. cruzi* both in vitro and in vivo. NO release upon reduction of the ruthenium nitrosyls in culture cells and animal models is thought to play an essential role in the antiproliferative and trypanocidal activities. Notably, *trans*-[Ru(NO)(NH_3_)_4_(imN)](BF_4_)_3_ allowed for survival of up to 80% of infected mice at a much lower dose (100 nmol/kg/day) than that required for benznidazole (385 μmol/kg/day) [203,204]. Ru(II) complexes with the formulae *cis*-[Ru(NO)(bpy)_2_(imN)](PF_6_)_3_ and *cis*-[Ru(NO)(bpy)_2_SO_3_]PF_6_ displayed inhibitory effects on the *T. cruzi* glyceraldehyde 3-phosphate dehydrogenase (GAPDH) (IC_50_ = 89 and 153 μM, respectively), which is a potential molecular target. These compounds exhibited in vitro and in vivo trypanocidal activities at doses up to 1000-fold lower than the clinical dose for benznidazole [205]. Furthermore, in a series of nitro/nitrosyl-Ru(II) complexes, *cis*, *trans*-[RuCl(NO)(dppb)(5,5’-mebipy)](PF_6_)_2_, where 5,5′-mebipy = 5,5′-dimethyl-2,2′-bipyridine and dppb = 1,4-*bis*(diphenylphosphino)butane, was the most active compound. The complex displayed an IC_50_ of 2.1 µM against trypomastigotes (the form of the parasite during the acute stage of the disease) and an IC_50_ of 1.3 µM against amastigotes (the form of the parasite during the chronic stage of the disease), while it was less toxic to macrophages. Moreover, the complex exerted synergistic activity with benznidazole in vitro against trypomastigotes and in vivo in infected mice [206].

**Figure 26:**
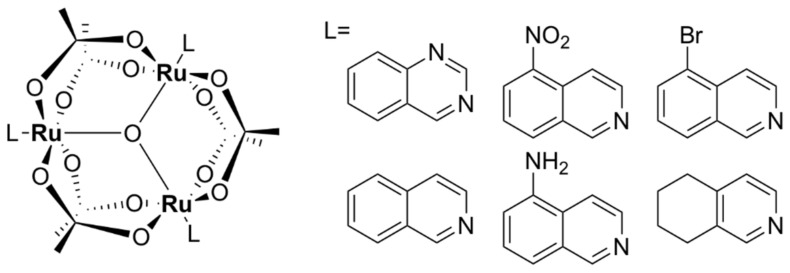
Chemical structures of symmetric trinuclear ruthenium complexes bearing azanaphthalene ligands with the general formula [Ru_3_O(CH_3_COO)_6_(L)_3_]PF_6_.

A series of symmetric trinuclear ruthenium complexes bearing azanaphthalene ligands with the general formula [Ru_3_O(CH_3_COO)_6_(L)_3_]PF_6_ (Figure 26), where L = (1) quinazoline (qui), (2) 5-nitroisoquinoline (5-nitroiq), (3) 5-bromoisoquinoline (5-briq), (4) isoquinoline (iq), (5) 5-aminoisoquinoline (5- amiq), and (6) 5,6,7,8-tetrahydroisoquinoline (thiq), were developed. All complexes presented in vitro trypanocidal activity, complex 6 being the lead compound of the series, with IC_50_ values of 1.39 µM against trypomastigotes and 1.06 µM against amastigotes. Complex 6 was up to 10 times more effective than benznidazole, while being essentially non-toxic to healthy mammalian cells (SI trypomastigote: 160, SI amastigote: 209) [207].

**Figure 27:**
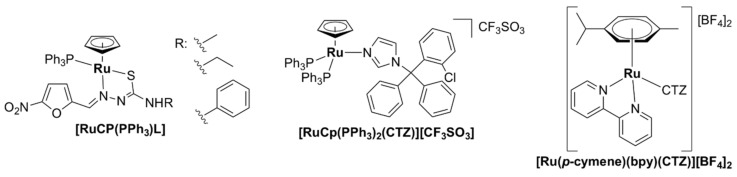
Chemical structures of Ru(II)–arene complexes with antitrypanosomal activity.

A range of Ru (II)–cyclopentadienyl thiosemicarbazone complexes displayed sub- or micromolar IC_50_ values against *T. cruzi* and *T. brucei*. Notably, [RuCp(PPh_3_)L] (Figure 27), where HL is the *N*-methyl derivative of 5-nitrofuryl containing thiosemicarbazone and Cp is cyclopentadienyl, exhibited high (IC_50_ *T. cruzi* = 0.41 µM; IC_50_ *T. brucei* = 3.5 µM) and selective activity (SI *T. cruzi* > 49 and SI *T. brucei* SI > 6). These complexes had the ability to interact with DNA in vitro, but no correlation with the biological activity was observed [208]. A Ru(II)–cyclopentadienyl clotrimazole complex, [RuCp(PPh_3_)_2_(CTZ)](CF_3_SO_3_) (Figure 27), where Cp = cyclopentadienyl and CTZ = clotrimazole, was more cytotoxic on *T. cruzi* than nifurtimox. With regard to its mechanism of action, the complex was shown to impair the sterol biosynthetic pathway in *T. cruzi* [209]. In another series of Ru(II)–cyclopentadienyl clotrimazole complexes, [Ru^II^(*p*-cymene)(bpy)(CTZ)][BF_4_]_2_ (Figure 27) was found to be the most active compound, increasing the activity of CTZ by a factor of 58 against *T. cruzi* (IC_50_ = 0.1 µM)*,* with no appreciable toxicity to human osteoblasts [210].

### 6.3. Antileishmaniasis Activity

Leishmaniasis is a disease caused by protozoan parasites of the genus *Leishmania* and is characterized by high morbidity. It is estimated that more than 1 billion people live in endemic areas, with more than 1 million new cases of leishmaniasis occurring annually. Current treatment for leishmaniasis relies on the use of pentavalent antimonials and other drugs, such as pentamidine isethionate, amphotericin B, and miltefosine. However, antileishmanial treatment cannot provide a sterile cure, and the parasite can cause a relapse when the human body is immunosuppressed [211,212].

An improved antiplasmodial activity in comparison with that of the free ligand has been reported for Ru(II)–lapachol complexes. [RuCl_2_(Lap)(dppb)] was active against *L. amazonensis* promastigotes and infected macrophages, with submicromolar IC_50_ values comparable with that of the reference drug, amphotericin B. In addition, the complex was not toxic to macrophages at concentrations much higher than the IC_50_ values [212].

[Ru^II^(*p*-cymene)(bpy)(CTZ)][BF_4_]_2_ (Figure 27) was found to be active against promastigotes of *L. major* at nanomolar concentrations (IC_50_ = 15 nM) and displayed no appreciable toxicity against human osteoblasts (SI > 500). Moreover, in *L. major*-infected mice macrophages, the complex caused a significant inhibition of the proliferation of intracellular amastigotes (IC_70_ = 29 nM) [210].

## 7. Antiviral Activity of Ruthenium Complexes

### 7.1. Anti-HIV Activity

The mixed-valent tetranuclear ruthenium–oxo oxalato cluster Na_7_[Ru_4_(µ_3_-O)_4_(C_2_O_4_)_6_] exerted promising anti-HIV-1 activity with over 98% inhibition of viral replication toward the R5-tropic HIV-1 strain at a 5 µM concentration and similar inhibitory activity toward X4-tropic viral replication. Moreover, the ruthenium–oxo oxalato cluster displayed selective anti-viral activity, with over 90% survival of the host cells registered at concentrations up to 50 µM. Notably, Na_7_[Ru_4_(µ_3_-O)_4_(C_2_O_4_)_6_] was 10-fold more effective against HIV-1 reverse transcriptase (IC_50_ = 1.9 nM) than the commonly used HIV-1 RT inhibitor 3’-azido-3’-deoxythmidine-5’-triphosphate (IC_50_ = 68 nM) [213].

Another ruthenium cluster, [H_4_Ru_4_(η^6^-*p*-benzene)_4_]^2+^, displayed selective activity against Polio virus, without inhibiting the growth of healthy human cells. It has been suggested that the complex might only be cytotoxic in Polio-infected cells, as the virus alters cell membrane permeability, facilitating passage for the cluster [94].

**Figure 28:**
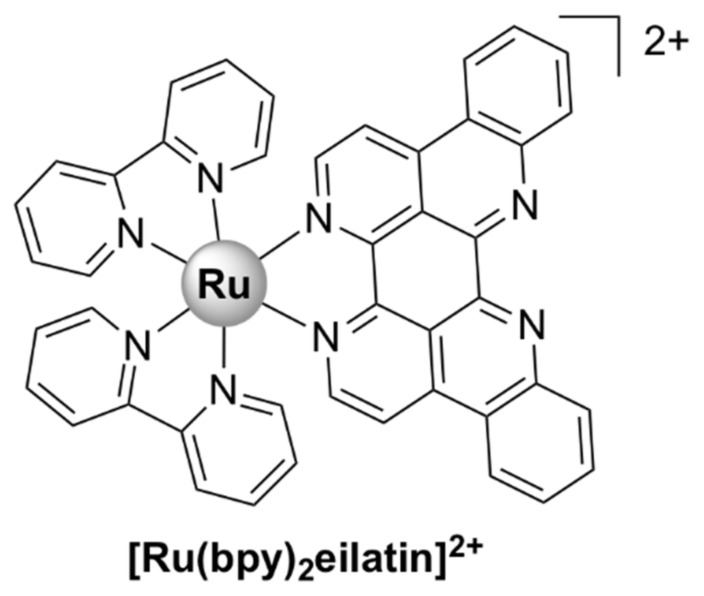
Chemical structure of [Ru(bpy)_2_eilatin]^2+^.

[Ru(bpy)_2_eilatin]^2+^ (Figure 28), where eilatin = dibenzotetraazaperylene, inhibited HIV-1 replication in CD4+ HeLa cells and in human peripheral blood monocytes (IC_50_ values ~1 µM). Eilatin is a fused, heptacyclic aromatic alkaloid that was isolated from the sea squirts belonging to *Eudistoma* sp., reported to possess cytotoxic and antiproliferative activities. [Ru(bpy)_2_eilatin]^2+^ is a kinetically inert complex and in vitro studies suggest that its mechanism of action relies upon inhibition of key protein–RNA interactions. The planar structure of the bidentate ligand, eilatin, was found to be essential for the activity of the complex [214].

### 7.2. Anti-SARS-Cov-2 Activity

In spite of the extensive vaccination campaigns that are currently ongoing, the severe acute respiratory syndrome coronavirus 2 (SARS-CoV-2) is spreading at an alarming pace across the world. The large number of mutations rendered several new variants less susceptible to treatment options, and possibly to vaccines. Thus, there is still an urgent need for the development of new drugs with a broader spectrum of activity [215,216].

**Figure 29:**
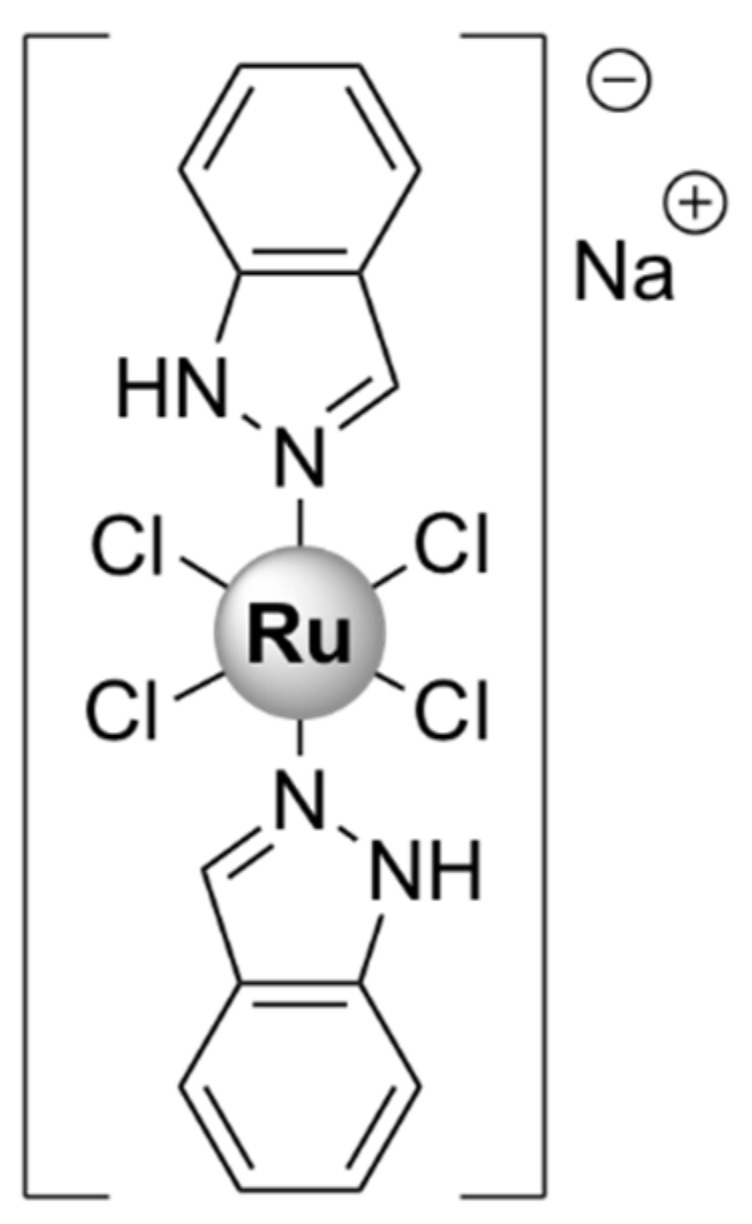
Chemical structure of BOLD-100 (sodium trans-[tetrachlorobis(1H-indazole)ruthenate(III)], KP1339).

BOLD-100 (sodium trans-[tetrachlorobis(1H-indazole)ruthenate(III)], KP1339, Figure 29), developed as an anticancer agent, was shown to selectively inhibit stress-induced upregulation of 78-kDa glucose-regulated protein (GRP78) [217,218,219], which is a master chaperone protein serving critical functions in the endoplasmic reticulum of normal cells [220,221]. The interaction of SARS-CoV-2 spike protein with the GRP78 protein located on the cell membrane can mediate viral entry. Therefore, disruption of this interaction may be used to develop novel therapeutic strategies against coronavirus [222]. Indeed, BOLD-100 was reported to reduce viral loads in various COVID-19 variants, including the more virulent B.1.1.7, originally identified in the United Kingdom. Unlike vaccines, which are more effective against certain viral variants, BOLD-100, with a broad antiviral mechanism of action, appears to remain active on all mutant strains [223]. In vivo studies are currently in progress.

BOLD-100 has a tolerable safety profile (minimal neurological or hematological effects), as was shown in a recently completed phase I clinical study involving 41 patients with advanced cancer. Moreover, it is currently undergoing clinical trials in combination with FOLFOX chemotherapy (which includes folinic acid, 5-fluorouracil, and oxaliplatin) for gastrointestinal solid tumors [224]. Therefore, BOLD-100 has already been successfully developed as a clinical-stage product, which suggests its potential for rapid further clinical development against COVID-19.

Additionally, [Ru(bpy)_3_]^2+^ is used in the Elecsys^®^ Anti-SARS-CoV-2, a chemiluminescence immunoassay intended for qualitative detection of antibodies to SARS-CoV-2 in human serum and plasma, which has been approved worldwide. In this assay, the SARS-CoV-2-specific recombinant antigen is labeled with the ruthenium complex [225,226]. Other metal complexes identified as potential anti-SARS-Cov-2 agents include auranofin [227,228] and Re(I) tricarbonyl complexes [229].

## 8. Conclusions

Ruthenium-based antimicrobial agents have a fairly complex mode of action involving multiple mechanisms acting in synergy. The knowledge gained so far in this area suggests that the activity of ruthenium compounds against microbial cells is based upon their ability to induce oxidative stress, interact with the genetic material, proteins, or other intracellular targets, and/or damage the cell membranes. The complex interplay between these modes of action is likely responsible for the activity of some ruthenium-based compounds against drug-resistant strains.

Generally, ruthenium complexes exert excellent activity against Gram-positive bacteria (e.g., *S. aureus* and MRSA) and, with some exceptions (see, for instance, the dinuclear polypyridylruthenium(II) complexes and ruthenium-based CORMs), display lower activity towards Gram-negative strains (e.g., *E. coli* and *P. aeruginosa*). With regard to their activity against Gram-negative bacteria, one can notice a trend towards higher efficacy against *E. coli* when compared with *P. aeruginosa* and *K. pneumoniae*. For most classes of compounds, activity towards both Gram-negative and Gram-positive strains has been correlated to the uptake of the complex into the cells.

Additionally, this work highlights recent advances in ruthenium-based compounds that are active against neglected tropical diseases caused by parasites, such as malaria, Chagas’ disease, and leishmaniasis. Notably, several complexes possess excellent activity, at submicromolar concentrations, results that raise awareness about the potential use of ruthenium compounds as effective antiparasitic agents. Moreover, the antiviral activity of ruthenium complexes, particularly the anti-HIV and anti-SARS-Cov-2 activities, has been reviewed herein. It is worth noting that BOLD-100 (formerly denoted KP1339) displays a broad antiviral mechanism of action and appears to remain active on all mutant strains of the SARS-Cov-2 virus.

In general terms, ruthenium complexes have been shown to display low levels of toxicity towards healthy eukaryotic cells in vitro and in vivo. This finding underlines the potential of these compounds for future clinical development, since selective toxicity against microbial over host cells in vitro and in vivo is imperative for a potential drug to advance in clinical trials. More in vivo studies are clearly needed in order to provide proof beyond a reasonable doubt that ruthenium complexes are strong candidates in the field of antimicrobial drug discovery.

In conclusion, this work aimed to highlight the potential of ruthenium-based compounds as novel antimicrobial agents due to the diverse range of complex 3D structures and modes of action they provide. Given that the pipeline of new antibiotics is running dry, the ruthenium species with high activity and selectivity presented herein may represent the starting point for a much-needed new class of antimicrobial agents. Therefore, we hope that this review will succeed in raising awareness about the potential of ruthenium complexes for antimicrobial applications and spur further research into their development.

## Figures and Tables

**Table 1:** Activities of selected ruthenium complexes against bacteria, toxicity to healthy mammalian cells, and mode of action.

Complex [Reference]	Activity Strain: MIC Values (µg/mL)		Toxicity to Healthy Mammalian Cells (IC_50_, µg/mL, 24 h, unless Stated Otherwise)	Modes of Action
	Gram-Positive Strains	Gram-Negative Strains		
*Polypyridylruthenium (II) complexes*				
[Ru(2,9-Me_2_phen)_2_(dppz)]^2+^ [40]	*S. aureus* MRSA252: 2, MRSA41: 4, MSSA160: 8, *B. subtilis* 168: 4	Not active on *E. coli* MC4100	-	bactericidal; DNA intercalation
R-825 [41]	*S. pneumoniae* D39 WT: 27.5 *piu*A mutant: 55	-	Not toxic to human alveolar epithelial A549 cells up to 480 µM	interference with iron acquisition systems in *S. pneumoniae* cells
X-03 [42]	*S. pneumoniae* D39: 25, *Streptococcus suis* 05ZYH33: 100, *S. pyogenes* MGAS5005: 25, *Listeria monocytogenes* 19,117: 25, *S. aureus* 29,213: 50	*E. coli* K12: > 200, *Vibrio alginolyticus* V12G01: > 200, *Vibrio parahaemolyticus* RIMD 2,210,633: > 200, *A. baumanii* 19,606: > 200	Not toxic to human alveolar A549 and bronchial HBE epithelial cells up to 100 µg/mL	interference with iron acquisition systems in *S. pneumoniae* cells; oxidative stress, membrane damage
[Ru(bpy)_2_Cl(clbzpy)]^+^ [43]	*S. aureus* ATCC 25,923: 500, *S. epidermidis* ATCC 12,228: 250	*P. aeruginosa* ATCC 10,145: not active	-	membrane damage
[Ru(bpy)_2_(methionine)]^2+^ [44]	upon blue LED irradiation *S. aureus* ATCC 25,923: 62.5, *S. epidermidis* ATCC 12,228: 125	*P. aeruginosa* ATCC 10,145: not active *E. coli* ATCC 11,303: 500	-	DNA photodamage
[Ru(dmb)_2_(ETPIP)]^2+^ [45]	*S. aureus Newman*: 50	-	-	-
[Ru(phen)_2_(ETPIP)]^2+^ [45]	*S. aureus Newman*: 25	-	-	inhibits biofilm formation; interacts with intracellular thiols
[Ru(bpy)_2_(BTPIP)]^2+^ [46]	*S. aureus Newman*: 16	-	-	inhibits biofilm formation
[Ru(bpy)_2_curcumin]^+^ [47]	*S. aureus* ATCC 29,213: 1	*A. baumanii* BAA-1605: > 64, *E. coli* ATCC 25,922: > 64, *K. pneumoniae* BAA-1705: > 64, *P. aeruginosa* ATCC 27,853: > 64	Vero (African green monkey kidney epithelial) cells: > 80	bactericidal; inhibits biofilm formation
[Ru(phen)_2_curcumin]^+^ [47]	*S. aureus* ATCC 29,213: 1	*A. baumanii* BAA-1605: 8–16, *E. coli* ATCC 25,922: > 64, *K. pneumoniae* BAA-1705: > 64, *P. aeruginosa* ATCC 27,853: > 64	Vero (African green monkey kidney epithelial) cells: > 80	-
Mono-bb_7_ [38]	*S. aureus* MSSA ATCC 25,923: 4 MRSA (JCU culture collection): 16	*E. coli* ATCC 25,922: 16 *P. aeruginosa* ATCC 27,853: > 128	-	bactericidal; membrane damage
Mono-bb_10_ [37,38]	*S. aureus* MSSA ATCC 25,923: 4 MRSA (JCU culture collection): 16	*E. coli* ATCC 25,922: 16 *P. aeruginosa* ATCC 27,853: 32	-	bactericidal
Mono-bb_16_ [37]	*S. aureus* MSSA ATCC 25,923: 16 MRSA (JCU culture collection): 16	*E. coli* ATCC 25,922: 64 *P. aeruginosa* ATCC 27,853: 64	-	-
*cis*-α-[Ru(phen)bb_12_]^2+^ [48]	*S. aureus* MSSA ATCC 25,923: 0.5 MRSA (JCU culture collection): 4	*E. coli* ATCC 25,922: 8 *P. aeruginosa* ATCC 27,853: 8	-	DNA binding
*cis*-β-[Ru(phen)(bb_12_)]^2+^ [48]	*S. aureus* MSSA ATCC 25,923: 0.5 MRSA (JCU culture collection): 4	*E. coli* ATCC 25,922: 16 *P. aeruginosa* ATCC 27,853: 32	-	DNA binding
[Ru(bb_7_)(dppz)]^2+^ [49]	*S. aureus* SH 1000: 2 MRSA USA 300 LAC JE2: 2	*E. coli* avian pathogenic: 8 uropathogenic: 8 *E. coli* MG1655: 8 *P. aeruginosa* PAO1: 16	human embryonic kidney HEK-293 cells: 27 (48 h), human fetal hepatocyte L02 cells: 64 (48 h)	bactericidal, DNA binding
[Ru(Me_4_phen)_2_(dppz)]^2+^ [50]	*S. aureus* SH1000: 9.7, *E. faecalis* V583: 38.8	*E. coli* MG1655: 4.9, EC958: 4.9, *P. aeruginosa* PA2017: 9.7 *A. baumannii* AB184: 9.7	-	bactericidal, chromosomal DNA binding
SCAR4 [51]	*M. tuberculosis* H_37_Rv ATCC 27,294 (neither G+, nor G-): 0.63	-	Mouse monocyte macrophage J774A.1 cell line: 19.5	covalent binding to DNA
SCAR5 [51]	*M. tuberculosis* H_37_Rv ATCC 27,294 (neither G+, nor G-): 0.26	-	J774A.1: 3.9	covalent binding to DNA
SCAR6 [51]	*M. tuberculosis* H_37_Rv ATCC 27,294 (neither G+, nor G-): 3.90	-	J774A.1: 78.2	covalent binding to DNA
RuNN [52]	*S. aureus* ATCC 25,923: 15.6, *S. aureus* ATCC 700,698 (MRSA): 62.5 *S. epidermidis* ATCC 12,228: 31.2, *S. epidermidis* ATCC 358,983: 62.5	-	no cytotoxic effect against human erythrocytes	bactericidal; inhibits biofilm formation
[Ru(hexpytri)_3_](PF_6_)_2_ [53]	*S. aureus* MSSA ATCC 25,923: 8, *S. aureus* MSSA NZRM 9653: 1, *S. aureus* MRSA MR 9519: 4, *S. pyogenes*: 4	*E. coli* ATCC 25,922: non-active	Vero cells: IC_50_ > 128 (48h)	cell wall/cytoplasmic membrane damage
[Ru(hexyltripy) (heptyltripy)]Cl_2_ [54]	*S. aureus* ATCC 25,923: 2	*E. coli* ATCC 25,922: 8	HDFa (skin cells): 16.4	abnormal cellular division
ΔΔ-Rubb_7_ [37,38]	*S. aureus* MSSA ATCC 25,923: 16 MRSA (JCU culture collection): 16	*E. coli* ATCC 25,922: 16 *P. aeruginosa* ATCC 27853: 128	Red blood cells: > 1024	bactericidal; membrane damage, interaction with ribosomal RNA
ΔΔ-Rubb_12_ [55,56]	*S. aureus* MSSA ATCC 25,923: 1 MRSA (JCU culture collection): 1	*E. coli* ATCC 25,922: 2 *P. aeruginosa* ATCC 27,853: 16	Baby hamster kidney (BHK): 113.9, HEK-293: 82.2	bactericidal; membrane damage, interaction with ribosomal RNA
ΔΔ-Rubb_16_ [56]	*S. aureus* MSSA ATCC 25,923: 1 MRSA (JCU culture collection): 1	*E. coli* ATCC 25,922: 4 *P. aeruginosa* ATCC 27,853: 8	Red blood cells: 22, BHK: 49.8, HEK-293: 35.1	bactericidal; membrane damage, interaction with ribosomal RNA
[Ru_2_(Me_4_phen)_2_(tpphz)]^4+^ [57,58,59]	*S. aureus* MSSA SH1000: 86, *Enterococcus faecalis* V583: 1	*E. coli* WT G1655: 2.5, EC958 ST131 (multi-drug-resistant, clinical isolate): 3.5, *P. aeruginosa* (clinical isolate): 4, *K. pneumoniae* (clinical isolate): 3.5, *A. baumannii* (clinical isolate): 3.5	HEK-293: 270	membrane and DNA damage
Cl-Rubb_7_-Cl [55,60]	*S. aureus* MSSA ATCC 25,923: 8 MRSA (JCU culture collection): 8	*E. coli* ATCC 25,922: 8 *P. aeruginosa* ATCC 27,853: 32	-	bactericidal
Cl-Rubb_12_-Cl [55,60]	*S. aureus* MSSA ATCC 25,923: 1 MRSA (JCU culture collection): 1	*E. coli* ATCC 25,922: 2 *P. aeruginosa* ATCC 27,853: 8	-	bactericidal
Cl-Rubb_16_-Cl [55,60]	*S. aureus* MSSA ATCC 25,923: 8 MRSA (JCU culture collection): 8	*E. coli* ATCC 25,922: 8 *P. aeruginosa* ATCC 27,853: > 128	-	bactericidal
Rubb_7_-Cl [56]	*S. aureus* MSSA ATCC 25,923: 8 MRSA (JCU culture collection): 16	*E. coli* ATCC 25,922: 1 *P. aeruginosa* ATCC 27,853: 16	BHK: 337.5, HEK-293: 98	interaction with chromosomal DNA and ribosomal RNA
Rubb_12_-Cl [56]	*S. aureus* MSSA ATCC 25,923: 1 MRSA (JCU culture collection): 1	*E. coli* ATCC 25,922: 1 *P. aeruginosa* ATCC 27,853: 16	BHK: 70.6, HEK-293: 87.3	interaction with chromosomal DNA and ribosomal RNA
Rubb_16_-Cl [56]	*S. aureus* MSSA ATCC 25,923: 1 MRSA (JCU culture collection): 2	*E. coli* ATCC 25,922: 4 *P. aeruginosa* ATCC 27,853: 64	BHK: 34.9, HEK-293: 63.5	interaction with chromosomal DNA and ribosomal RNA
Rubb_7_-tri [37,61]	*S. aureus* MSSA ATCC 25,923: 4 MRSA (JCU culture collection): 4	*E. coli* ATCC 25,922: 16 *P. aeruginosa* ATCC 27,853: 2	-	interaction with DNA
Rubb_7_-tetra (Rubb_7_-TL) [62]	*S. aureus* MSSA ATCC 25,923: 8 MRSA (JCU culture collection): 16	*E. coli* avian pathogenic: 16 uropathogenic: 16 *E. coli* MG1655: 16 *P. aeruginosa* PAO1: 32	BHK: 176 (24 h) BHK: 36.4 (72 h)	interaction with proteins
Rubb_7_-TNL [62]	*S. aureus* MSSA ATCC 25,923: 4 MRSA (JCU culture collection): 8	*E. coli* avian pathogenic: 16 uropathogenic: 16 *E. coli* MG1655: 8 *P. aeruginosa* PAO1: 16	BHK: 276 (24 h) BHK: 81.6 (72 h)	interaction with proteins
Rubb_12_-tri [37,55,61]	*S. aureus*: 1** MRSA (JCU culture collection): 1	*E. coli*: 4 *P. aeruginosa*: 32	BHK: 50.9 (72 h), HEK-293: 21.8 (72 h)	bactericidal, interaction with DNA
Rubb_12_-tetra [37,55,61]	*S. aureus*: 2** MRSA (JCU culture collection): 2	*E. coli*: 2 *P. aeruginosa*: 16	BHK: 43.7 (72 h), HEK-293: 21.3 (72 h)	bactericidal, interaction with DNA
Rubb_16_-tri [37,55,61]	*S. aureus*: 2** MRSA (JCU culture collection): 2	*E. coli*: 8 *P. aeruginosa*: 32	BHK: 25.1 (72 h), HEK-293: 20.2 (72 h)	bactericidal, interaction with DNA
Rubb_16_-tetra [37,55,61]	*S. aureus*: 2** MRSA (JCU culture collection): 2	*E. coli*: 8 *P. aeruginosa*: 32	BHK: 19.8 (72 h), HEK-293: 15.8 (72 h)	bactericidal, interaction with DNA
*Ruthenium-based CORMs*				
CORM-2 [63,64,65]	Growth inhibitory effects on *S. aureus* (MIC value not reported)	*E. coli* avian pathogenic: 250, uropathogenic: 250, *E. coli* MG1655: 250, *P. aeruginosa* PAO1: 3.8 *H. pylori* strains (including antibiotic resistant): 100–200	Murine RAW264.7 monocyte macrophages: > 50 (DMEM culture medium)	Bactericidal, inhibition of aerobic respiration, inhibition of biofilm formation and disruption of mature biofilms, ROS generation, interaction with chromosomal DNA and intracellular proteins, interference with iron homeostasis
CORM-3 [64,66,67]	Growth inhibitory effects on *S. aureus*, *Lactobacillus lactis* (MIC value not reported)	*E. coli* MG1655: 4 (minimal GDMM medium) and > 512 (in rich MH-II medium) *H. pylori* 26,695: 420 (antibiotic resistant strains)	L929 murine fibroblast cells: 63 (RPMI culture medium), RAW264.7: > 30 (DMEM culture medium)	
*Ruthenium complexes in Antimicrobial Photodynamic Therapy*				
[Ru(dmob)_3_]^2+^ [68]	*S. aureus* NCTC 10788: 12.5	*P. aeruginosa* NCTC 8626: 50	-	Light activation
*cis*-[Ru(bpy)_2_(INH)_2_]^2+^ [69]	*Mycobacterium smegmatis*: 4		human lung fibroblast MRC-5 cell line: > 200	465 nm blue light activation
[Ru(Ph_2_phen)_2_(dpp) PtCl_2_]^2+^ [70]	-	*E. coli* JM109: 8	-	visible light activation, binding to chromosomal DNA
[Ru(CO)_2_Cl_2_]_n_ [71]	*S. aureus CETC 240, coincident with ATCC 6538 P*: 0.033	*E. coli* CET 516, coincident with ATCC 8739: 0.0066	human dermal fibroblasts hDF: > 3.33	365 nm UV light activation, ROS generation, biofilm inhibition
